# Supplementary material for: Pre-liver transplant assessment of patients with acute-on-chronic liver failure: An international survey
Source: JHEP Rep. 2026 Feb 26;8(8):101794. doi: 10.1016/j.jhepr.2026.101794 (PMC13352056; doi:10.1016/j.jhepr.2026.101794)
Supplement: Multimedia component 1 [file mmc1.pdf]

Pre-liver transplant assessment of patients with acute-on-chronic liver failure: An international survey

Claire Bellec, Anéa Augé, Sébastien L'Hermite, Romain Moirand, Armand Abergel, Matthew Amstrong, Teresa Antonini, Rodolphe Anty, Marion Khaldi, Aurore Baron, Mouni Bensenane-Oussalah, William Bernal, Christophe Bureau, Nicolas Carbonell, Jean-François Cadranel, Filipe Cardoso, Paul Carrier, Audrey Coilly, Isabel Conde, Filomena Conti, Agnes Bonadona, Gonzalo Crespo, Sarwa Darwish Murad, Sébastien Dharancy, Christophe Duvoux, Cornelius Engelmann, François Faitot, Claire Francoz, Armand Garioud, René Gerolami, Elia Gigante, Odile Goria, Thierry Gustot, Brian Hogan, Ludovic Lagin, Adrien Lannes, Marianne Latournerie, Victor de Ledinghen, Giulia Magini, Martin Mateo, Arnaud Maurin, Manuela Merli, Magdalena Meszaros, Georges-Philippe Pageaux, Claire Perignon, Giovanni Perricone, Michael Praktiknjo, Noemi Reboux, Thomas Reiberger, Valentin Rolle, Isabelle Rosa , Ruxandra Sarba, Faustine Wartel, Delphine Verhoeven Weil, Alberto Zanetto, Laure Elkrief, Florent Artru

Table of contents

Table S1……………………………………………………………………………………….2

Table S2……………………………………………………………………………………….4

Table S3……………………………………………………………………………………...10

Table S4……………………………………………………………………………………...16

Table S1. List of the participating centers.

	Country	Transplant / non transplant center	Academic / Non academic hospital
Clermont-Ferrand University Hospital	France	Transplant	Academic
Hospices civils de Lyon, Lyon University Hospital	France	Transplant	Academic
Pasteur Hospital of Nice, Nice University Hospital	France	Transplant	Academic
Pierre-Paul Riquet Hospital of Toulouse, Toulouse University Hospital	France	Transplant	Academic
Paul Brousse Hospital, Paris-Saclay University	France	Transplant	Academic
Pitié Salpétrière Charles Foix University Hospital	France	Transplant	Academic
Michallon Hospital, Grenoble Alpes University Hospital	France	Transplant	Academic
Bordeaux University Hospital	France	Transplant	Academic
Lille University Hospital	France	Transplant	Academic
Strasbourg University Hospital	France	Transplant	Academic
Beaujon Hospital, Paris Cité University	France	Transplant	Academic
Marseille Timone University Hospital	France	Transplant	Academic
Montpellier University Hospital	France	Transplant	Academic
Jean Minjoz Hospital, Besançon University Hospital	France	Transplant	Academic
Trousseau Hospital, Tours University Hospital	France	Transplant	Academic
Pontcahillou Hospital, Rennes University Hospital	France	Transplant	Academic
Hôtel Dieu Hospital, Nantes University Hospital	France	Non transplant	Academic
Saint Antoine Hospital, Sorbonne University	France	Non transplant	Academic
Dupuytren Hospital, Limoges University Hospital	France	Non transplant	Academic
Christian Cabrol Hospital, Reims University Hospital	France	Non transplant	Academic
Angers University Hospital	France	Non transplant	Academic
François-Mitterand Hospital, Dijon University Hospital	France	Non transplant	Academic
Avicenne Hospital, Bobigny University	France	Non transplant	Academic
Côte de Nacre Hospital, Caen Normandie University Hospital	France	Non transplant	Academic
Cavale Blanche Hospital, Brest University Hospital	France	Non transplant	Academic
Poitiers University Hospital	France	Non transplant	Academic
Amiens-Picardie University Hospital	France	Non transplant	Academic
Charles-Nicolle Hospital, Rouen University Hospital	France	Non transplant	Academic

Henri-Mondor Hospital, Paris-Est Créteil University	France	Non transplant	Academic
Brabois Hospital, Nancy University Hospital	France	Non transplant	Academic
Créteil Intercommucal Hospital	France	Non transplant	Non academic
Sud Francilien Hospital, Corbeil-Essonnes	France	Non transplant	Non academic
Sud de l'Oise Public Hospital Group, Creil	France	Non transplant	Non academic
Villeuneuve-Saint-Georges Intercommunal Hospital	France	Non transplant	Non academic
Valenciennes Hospital	France	Non transplant	Non academic
Le Mans Hospital	France	Non transplant	Non academic
Départemental Vendée Hospital , La Roche sur Yon	France	Non transplant	Non academic
University Hospital Birmingham	United Kingdom	Transplant	Academic
Medical University of Vienna	Austria	Transplant	Academic
King’s College Hospital, London	United Kingdom	Transplant	Academic
Curry Cabral Hospital, Lisbon	Portugal	Transplant	Academic
Hospital Universitario y Politecnico La Fe, Valence	Spain	Transplant	Academic
Hospital Clínic de Barcelona	Spain	Transplant	Academic
Charité - Universitaetsmedizin Berlin	Germany	Transplant	Academic
Erasme Hospital, Bruxelles University Hospital	Belgium	Transplant	Academic
Royal Free Hospital, London	United Kingdom	Transplant	Academic
Geneve University Hospital	Switzerland	Transplant	Academic
Ramón y Cajal University Hospital, Madrid	Spain	Transplant	Academic
Rome University Hospital	Italy	Transplant	Academic
Erasmus MC University Medical , Rotterdam	Netherlands	Transplant	Academic
ASST Grande Ospedale Metropolitano Niguarda, Milan	Italy	Transplant	Academic
Münster University Hospital	Germany	Transplant	Academic
Padua University Hospital	Italy	Transplant	Academic

Table S2: Answers to the questionnaire from the 53 centers according to patient's setting at the time of pre-transplant evaluation.

Question	Outpatients (OutPat)	Patients hospitalized in liver ward without ACLF (Hosp)	Patients with ACLF in ICU (ACLF-ICU)	p value ACLF *vs.* OutPat	p value ACLF *vs.* Hosp
**Cardiac and respiratory assessment**					
**Do you systematically perform an ECG?**					
Yes	49 (92.5)	52 (98.1)	52 (98.1)	0.17	1
No	4 (7.5)	1 (1.9)	1 (1.9)		
**Do you systematically perform a TTE?**					
Yes	53 (100)	52 (98.1)	53 (100)	1	0.32
No	0 (0)	1 (1.9)	0 (0)		
**Do you systematically request a stress exam?**					
Yes	11 (20.8)	9 (17.0)	1 (1.9)	0.002	0.008
No	42 (79.2)	44 (83.0)	52 (98.1)		
**Do you perform a stress exam in case of cardiovascular risk factors?**					
Yes	22 (41.5)	24 (45.3)	5 (9.4)	0.0002	<0.0001
No	31 (58.5)	29 (54.7)	48 (90.6)		
**Do you systematically perform a CCTA or a CACS?**					
Yes	4 (7.5)	3 (5.7)	0 (0)	0.04	0.08
No	49 (92.5)	50 (94.3)	53 (100)		
**Do you perform a CCTA or a CACS in case of cardiovascular risk factors?**					
Yes	11 (20.8)	11 (20.8)	2 (3.8)	0.008	0.008
No	42 (79.2)	42 (79.2)	51 (96.2)		
**When do you perform a coronarography?**					
- In case of cardiovascular risk factors	18 (34.0)	16 (30.2)	12 (22.6)	0.19	0.38
- In case of symptoms	27 (50.9)	23 (43.4)	31 (58.5)	0.43	0.12
- In case of abnormal noninvasive tests	40 (75.5)	35 (66.0)	22 (41.5)	0.0004	0.01
**What percentage of patients undergo a right-heart catheterism?**					

- < 25% patients<br>- > 25% of patients	49 (92.5)<br>4 (7.5)	47 (88.7)<br>6 (11.3)	51 (96.2)<br>2 (3.8)	0.41	0.17
**When do you perform a right-heart catheterism in your center?**					
- Always	2 (3.8)	2 (3.8)	1 (1.9)	0.56	0.56
- In case of abnormal TTE results	51 (96.2)	50 (94.3)	51 (96.2)	1	0.65
- In case of symptoms	17 (32.1)	16 (30.2)	10 (18.9)	0.12	0.18
**When do you perform a doppler of supra-aortic trunks?**					
- Always<br>- In case of risk factors<br>- Never	20 (37.8)<br>16 (30.2)<br>8 (15.1)	23 (43.4)<br>26 (49.1)<br>19 (35.8)	10 (18.9)<br>11 (20.8)<br>26 (49.1)	0.008	<0.0001
**When do you request a cardiologist consultation?**					
- Always<br>- In case of risk factors<br>- Never	27 (50.9)<br>23 (43.4)<br>3 (5.7)	24 (45.3)<br>24 (45.3)<br>5 (9.4)	17 (32.1)<br>19 (35.8)<br>17 (32.1)	0.002	<0.0001
**What respiratory exam(s) do you systematically request?**					
- Thoracic CT scan	49 (92.5)	50 (94.3)	53 (100)	0.04	0.08
- Respiratory function test	47 (88.7)	40 (75.5)	13 (24.5)	<0.0001	<0.0001
- 6 minutes' walk test	7 (13.2)	4 (7.5)	0 (0)	0.006	0.04
- pneumologist consultation	10 (18.9)	8 (15.1)	3 (5.7)	0.04	0.11
**Addictology assessment**					
**How many patients are assessed by a senior addictologist?**					
- < 25% patients<br>- 26-50% patients<br>- 51-75% patients<br>- > 75% patients	3 (5.7)<br>9 (17.0)<br>8 (15.1)<br>33 (62.3)	5 (13.5)<br>2 (5.4)<br>4 (10.8)<br>26 (70.3)	8 (21.6)<br>4 (10.8)<br>6 (16.2)<br>19 (51.4)	0.01	0.39
**If the first addictology assessment was realized in a nontransplant center, is it systematically performed again in the transplantation center?**					
- Yes	38 (71.7)	38 (71.7)	36 (67.9)	0.67	0.67

- No	15 (28.3)	15 (28.3)	17 (32.1)		
**Apart from alcohol-associated hepatitis, what is the minimal required duration of alcohol cessation to consider liver transplantation?**					
- ≥ 6 months	16 (30.1)	8 (15.2)	5 (9.4)		
- 3-6 months	19 (35.9)	20 (37.7)	12 (22.6)		
- 1-3 months	1 (1.9)	4 (7.5)	2 (3.8)	0.008	<0.0001
- < 1 month	0	0	1 (1.9)		
- Case by case decision	17 (32.1)	21 (39.6)	33 (62.3)		
**Decisive components to predict alcohol relapse risk after transplantation? (1- most important, 8- less important)**					
- Addiction diagnosis according to DSM V	4 (1-8)	5 (2-8)	5 (2-8)	0.73	0.92
- Former failure of withdrawal cure	3 (2-6)	3 (2-6)	3 (2-5)	0.29	0.43
- Polyaddiction	4 (2-6)	4 (2-7)	4 (3-6)	0.45	0.45
- Psychiatric comorbidities	4 (3-7)	4 (2-6)	4 (3-6)	0.45	0.88
- Socio-professional insertion	5 (4-6)	5 (4-5)	4 (3-5)	0.36	0.75
- Relatives' quality	5 (4-6)	5 (4-6)	5 (3-6)	0.96	0.79
- Insight of their alcohol use disorder	3 (2-6)	4 (2-7)	5 (3-7)	0.17	0.63
- Addictologist follow-up	6 (3-7)	6 (3-7)	6 (2-7)	0.90	0.98
- **Which score(s) do you routinely use for your addictology assessment?**					
- AUDIT score	47 (88.7)	45 (84.9)	47 (88.7)	1.0	0.57
- HRAR score	7 (13.2)	9 (17.0)	8 (15.1)	0.78	0.79
- SALT score	5 (9.4)	7 (13.2)	6 (11.3)	0.75	0.77
**In which circumstances do you support your addictology assessment by questioning the relatives?**					
- Always	32 (60.4)	37 (69.8)	38 (71.7)	0.22	0.81
- In case of patient's encephalopathy/intubation	17 (32.1)	13 (24.5)	18 (34.0)	0.84	0.29
**Who is questioned in the family when this is done?**					
- The partner	53 (100)	53 (100)	52 (98.1)	0.31	0.31
- Children and parents	45 (84.9)	49 (92.5)	50 (94.3)	0.11	0.70

- Friends /colleagues<br>- The general practitioner	4 (7.5)<br>34 (64.1)	5 (9.4)<br>32 (60.4)	8 (15.1)<br>37 (69.8)	0.22<br>0.54	0.37<br>0.31
**How often do you perform blood, urinary or capillary screening tests?**<br>- < 25% patients<br>- 26 -50% patients<br>- 51 -75% patients<br>- > 75% patients	18 (34.0)<br>10 (18.8)<br>7 (13.2)<br>18 (34.0)	23 (43.4)<br>5 (9.4)<br>8 (15.1)<br>17 (32.1)	31 (58.5)<br>6 (11.3)<br>2 (3.8)<br>14 (26.4)	0.052	0.16
**Which biological or urinary test is most often performed in your center?**<br>- Carboxydate Deficience Transferrine<br>- Phosphatydyléthanol<br>- Urinary ethyl glucuronide<br>- Capillary ethyl glucoronide	15 (28.3)<br>20 (37.7)<br>14 (26.4)<br>4 (7.5)	14 (26.4)<br>19 (35.9)<br>17 (32.1)<br>3 (5.7)	17 (32.1)<br>17(32.1)<br>16 (30.2)<br>3 (5.7)	0.89	0.77
**Do you always take into account the addictologist's opinion?**<br>- Yes<br>- No	43 (81.1)<br>10 (18.9)	43 (81.1)<br>10 (18.9)	42 (79.3)<br>11 (20.7)	0.81	0.81
**Oncologic assessment**<br>**In patients above 50 years old, what exams do you perform?**					
**When do you perform a colonoscopy**<br>- In all patients<br>- In case of risk factors of colorectal neoplasm	34 (64.1)<br>17 (32.1)	26 (49.1)<br>16 (30.2)	9 (16.7)<br>13 (24.5)	<0.0001<br>0.39	0.0004<br>0.51
**Do you systematically perform an upper GI endoscopy?**<br>- Yes<br>- No	49 (92.5)<br>4 (7.5)	43 (81.1)<br>10 (18.9)	34 (64.1)<br>19 (35.9)	0.0004	0.05
**Do you systematically perform a CT scan of the abdomen and pelvis?**<br>- Yes<br>- No	49 (92.5)<br>4 (7.5)	49 (92.5)<br>4 (7.5)	53 (100)<br>0 (0)	0.04	0.04
**Do you systematically perform a PSA dosage in men?**<br>- Yes	42 (79.3)	41 (77.4)	36 (67.9)	0.19	0.28

- No	11(20.7)	12 (22.6)	17 (32.1)		
**Do you systematically perform a mammography, pap smear, and gynecologic exam for women?**<br>- Yes<br>- No	 <br>43 (81.1)<br>10 (18.9)	 <br>35 (66.0)<br>18 (34.0)	 <br>16 (30.2)<br>37 (69.8)	 <br><0.0001	 <br>0.0002
**Do you systematically perform an ENT consultation?**<br>- Yes<br>- No	 39 (73.6)<br>14 (26.4)	 34 (64.1)<br>19 (35.9)	 19 (35.9)<br>34 (64.1)	 0.0001	 0.004
**Do you systematically perform a dermatology consultation?**<br>- Yes<br>- No	 25 (47.2)<br>23 (52.8)	 23 (43.4)<br>30 (56.6)	 9 (17.0)<br>44 (83.0)	 0.0009	 0.004
**Nutritional assessment**					
**When do you request nutritionist or dietitian consultation?**<br>- Systematically<br>- In case of clinical malnutrition<br>- Never	 16 (29.7)<br>37 (70.3)<br>0 (0)	 26 (49.1)<br>15 (28.3)<br>12 (22.6)	 19 (35.8)<br>15 (28.3)<br>19 (35.8)	 <br><0.0001	 <br>0.003
**When do you perform a screening for malnutrition using questionnaires such as SGA, RFH-NPT**<br>- Systematically<br>- In case of clinical malnutrition	 <br>10 (18.9)<br>16 (30.2)	 <br>13 (24.5)<br>11 (20.8)	 <br>9 (17.0)<br>8 (15.1)	 <br>0.80<br>0.06	 <br>0.34<br>0.45
**When do you perform a screening for sarcopenia using psoas measurement by CT scan?**<br>- Systematically<br>- In case of clinical malnutrition	 <br>4 (7.6)<br>7 (13.2)	 <br>4 (7.6)<br>6 (11.3)	 <br>5 (9.4)<br>8 (15.1)	 <br>1<br>0.78	 <br>0.73<br>0.57
**When do you perform a screening for frailty using dedicated tests (for example LFI)**?<br>- Systematically<br>- In case of clinical malnutrition	 14 (26.4)<br>23 (43.4)	 11 (20.8)<br>24 (45.3)	 8 (15.1)<br>6 (11.3)	 0.16<br>0.0002	 0.45<br>0.0001
**General considerations**					

**In your experience, what is the timeframe in days between initiation of LT assessment and listing?**	45 (30-90)	18 (14-30)	7 (3-10)	<0.0001	<0.0001
**What proportion of ACLF patients develop ACLF while already listed for LT, with a complete pre-transplant workup?**<br>- < 25% patients<br>- 26 -50% patients<br>- 51 -75% patients<br>- 75% patients			 38 (71.7)<br>11 (20.8)<br>3 (5.7)<br>1 (1.9)	N/A	N/A
**How do you describe the pre-transplant workup in ACLF patients in ICU, as compared with other situations?**<br>- Similar to other situations<br>- Abbreviated workup to allow for a quick listing assuming that it will still be able to identify definitive contraindications to LT<br>- Abbreviated workup to allow for a quick listing with the risk of not identifying definitive contraindications to LT<br>- More comprehensive workup to secure LT results in this very sick population			 1 (1.9)<br>39 (73.6)<br>12 (22.6)<br>1 (1.9)	N/A	N/A

Abbreviations : ACLF: Acute On Chronic Liver Failure, AUD: alcohol use disorder, AUDIT: Alcohol Use Disorders Identification Test, CCAS: coronary artery calcium scan, CCTA: coronary CT angiography, DSM: Diagnostic and Statistical Manual of Mental Disorders, ECG: electrocardiogram, EGD: oesophagograstroduodenoscopy, ENT: Ear-Nose-Throat, HRAR: High-Risk Alcoholism Relapse, Hosp: hospitalized patients without ALCF; LFI: liver frailty index, LT: liver transplantation, OutPat: outpatients, PSA: Prostatic Specific Antigen, RFH-NPT: Royal Free Hospital nutritional prioriziting tool, SALT: Sustained Alcohol Use After Early Liver Transplant, SGA: subjective global assessment, TAP: thoraco-abdomino-pelvic, TTE: trans-thoracic echocardiography

Table S3. Answers to the questionnaire regarding pre-transplant evaluation in ACLF-ICU patients among respondents from 32 liver transplant centers according to the country (French *vs.* other countries).

Question	French transplant centers	European transplant centers	p value
**Cardiac and respiratory assessment**			
**Do you systematically perform an ECG?**			
Yes	16 (100)	16 (100)	1
No	0 (0)	0 (0)	
**Do you systematically perform a TTE?**			
Yes	16 (100)	16 (100)	1
No	0 (0)	0 (0)	
**Do you systematically request a stress exam?**			
Yes			
No	0 (0)	0 (0)	1
	16 (100)	16 (100)	
**Do you perform a stress exam in case of cardiovascular risk factors?**			
Yes	0 (0)	3 (25.0)	0.07
No	16 (100)	13 (75.0)	
**Do you systematically perform a CCTA or a CACS?**			
Yes			
No	0 (0)	0 (0)	1
	16 (100)	16 (100)	
**Do you perform a CCTA or a CACS in case of cardiovascular risk factors?**			
Yes	0 (0)	2 (12.5)	0.14
No	16 (100)	14 (87.5)	
**When do you perform a coronary angiography?**			
- In case of cardiovascular risk factors	4 (25.0)	4 (25.0)	1
- In case of symptoms	10 (62.5)	4 (25.0)	0.03
- In case of abnormal noninvasive tests	4 (25.0)	11 (68.8)	0.01
**What percentage of patients undergo a right-heart catheterism?**			

- < 25% patients<br>- > 25% of patients	15 (93.8)<br>1 (6.2)	16 (100)<br>0 (0)	0.31
**When do you perform a right-heart catheterism in your center?**<br>- Always<br>- In case of abnormal TTE results<br>- In case of symptoms	 <br>0 (0)<br>15 (93.8)<br>2 (12.5)	 <br>0 (0)<br>16 (100)<br>2 (12.5)	 <br>1<br>0.31<br>1
**When do you perform a doppler of supra-aortic trunks?**<br>- Always<br>- In case of risk factors<br>- Never	 <br>2 (12.5)<br>4 (25.0)<br>10 (62.5)	 <br>1 (6.3)<br>7 (43.8)<br>8 (50.0)	0.50
**When do you request a cardiologist consultation?**<br>- Always<br>- In case of risk factors<br>- Never	 6 (37.5)<br>3 (18.8)<br>7 (43.7)	 5 (31.3)<br>10 (62.5)<br>1 (6.2)	0.02
**What respiratory exam(s) do you systematically request?**<br>- Thoracic CT scan<br>- Respiratory function test<br>- 6 minutes' walk test<br>- pneumologist consultation	 <br>16 (100)<br>2 (12.5)<br>0 (0)<br>1 (6.3)	 <br>16 (100)<br>3 (18.8)<br>0 (0)<br>1 (6.3)	 <br>1<br>0.62<br>1<br>1
**Addictology assessment**			
**How many patients are assessed by a senior addictologist?**<br>- < 25% patients<br>- 26-50% patients<br>- 51-75% patients<br>- > 75% patients	 <br>2 (12.5)<br>2 (12.5)<br>5 (31.3)<br>7 (43.7)	 <br>6 (37.5)<br>1 (6.3)<br>1 (6.3)<br>8 (50.0)	0.17
**If the first addictology assessment was realized in a nontransplant center, is it systematically performed again in the transplantation center?**<br>- Yes<br>- No	 <br><br>8 (50.0)<br>8 (50.0)	 <br><br>15 (93.8)<br>1 (6.2))	0.006

**Apart from alcohol-related hepatitis, what is the minimal required duration of alcohol cessation to consider liver transplantation?**			
- ≥ 6 months	1 (6.3)	3 (18.8)	
- 3-6 months	0 (0)	4 (25.0)	0.06
- 1-3 months	2 (12.5)	0 (0)	
- < 1 month	0 (0)	1 (6.3)	
- Case by case decision	13 (81.2)	8 (50.0)	
**Decisive components to predict alcohol relapse risk after transplantation? (1- most important, 8- less important)**			
- Addiction diagnosis according to DSM V	3 (1-8)	4 (3-8)	0.23
- Former failure of withdrawal cure	6 (5-8	3 (1-4)	0.0003
- Polyaddiction	6 (3-7)	4 (2-6)	0.07
- Psychiatric comorbidities	4 (3-7)	5 (3-6)	0.87
- Socio-professional insertion	4 (3-5)	6 5-8)	0.003
- Relatives' quality	5 (4-6)	6 (57)	0.12
- Insight of their alcohol use disorder	4 (1-7)	3 (2-6)	0.94
- Addictologist follow-up	5 (2-7)	5 (2-7)	0.78
- **Which score(s) do you routinely use for your addictology assessment?**			
- AUDIT score	14 (87.5)	14 (87.5)	1
- HRAR score	3 (18.8)	2 (12.5)	0.62
- SALT score	1 (6.3)	3 (18.8)	0.29
**In which circumstances do you support your addictology assessment by questioning the relatives?**			
- Always	15 (93.8)	10 (62.5)	0.03
- In case of patient's encephalopathy/intubation	1 (6.2)	8 (50.0)	0.006
**Who is questioned in the family when this is done?**			
- The partner	16 (100)	16 (100)	1
- Children and parents	16 (100)	14 (87.5)	0.14
- Friends /colleagues	4 (25.0)	2 (12.5)	0.37
- The general practitioner	13 (83.8)	6 (37.5)	0.01
**How often do you perform blood, urinary or capillary screening tests?**			
- < 25% patients	9 (56.3)	9 (56.3)	

- 26 -50% patients<br>- 51 -75% patients<br>- > 75% patients	3 (18.7)<br>0 (0)<br>4 (25.0)	2 (12.5)<br>1 (6.3)<br>4 (25.0)	0.75
**Which biological or urinary test is most often performed in your center?**<br>- Carboxydate Deficience Transferrine<br>- Phosphatydyléthanol<br>- Urinary ethyl glucuronide<br>- Capillary ethyl glucoronide	5 (31.2)<br>6 (37.5)<br>4 (25.0)<br>1 (6.3)	5 (31.2)<br>1 (6.3)<br>9 (56.3)<br>1 (6.3)	0.14
**Do you always take into account the addictologist's opinion?**<br>- Yes<br>- No	12 (75.0)<br>4 (25.0)	15 (93.8)<br>1 (6.2)	0.14
**Oncologic assessment**<br>**In patients above 50 years old, what exams do you perform?**			
**When do you perform a colonoscopy**<br>- In all patients<br>- in case of risk factors of colorectal neoplasm	1 (6.3)<br>4 (25.0)	2 (12.5)<br>4 (25.0)	0.54<br>1
**Do you systematically perform an upper GI endoscopy?**<br>- Yes<br>- No	11 (68.8)<br>5 (31.2)	7 (43.8)<br>9 (56.2)	0.15
**Do you systematically perform a CT scan of the abdomen and pelvis?**<br>- Yes<br>- No	16 (100)<br>0 (0)	16 (100)<br>0 (0)	1
**Do you systematically perform a PSA dosage in men?**<br>- Yes<br>- No	11 (68.8)<br>5 (31.2)	9 (56.3)<br>7 (43.7)	0.47
**Do you systematically perform a mammography, pap smear, and gynecologic exam for women?**<br>- Yes<br>- No	5 (31.2)<br>11 (68.8)	4 (25.0)<br>12 (75.0)	0.69
**Do you systematically perform an ENT consultation?**			

- Yes<br>- No	6 (37.5)<br>10 (62.5)	3 (18.8)<br>13 (81.2)	0.24
**Do you systematically perform a dermatology consultation?**<br>- Yes<br>- No	3 (18.8)<br>13 (81.2)	1 (6.3)<br>15 (93.7)	0.29
**Nutritional assessment**			
**When do you request nutritionist or dietitian consultation?**<br>- Systematically<br>- In case of clinical malnutrition<br>- Never	7 (43.7)<br>5 (31.3)<br>4 (25.0)	4 (25.0)<br>4 (25.0)<br>8 (50.0)	0.33
**When do you perform a screening for denutrition using questionnaires such as SGA, RFH-NPT**<br>- Systematically<br>- In case of clinical malnutrition	2 (12.5)<br>1 (6.3)	1 (6.3)<br>3 (18.8)	0.54<br>0.29
**When do you perform a screening for sarcopenia using psoas measurement by CT scan?**<br>- Systematically<br>- In case of clinical malnutrition	3 (18.8)<br>3 (18.8)	2(12.5)<br>2 (12.5)	0.63<br>0.63
**When do you perform a screening for frailty using dedicated tests (for example LFI)**?<br>- Systematically<br>- In case of clinical malnutrition	3 (18.8)<br>3 (18.8)	3 (18.8)<br>2 (12.5)	1<br>0.63
**General considerations**			
**In your experience, what is the timeframe in days between initiation of LT assessment and listing?**	5 (3-7)	5 (3-7)	7 (5-15)
**What proportion of ACLF patients develop ACLF while already listed for LT, with a complete pre-transplant workup?**<br>- < 25% patients<br>- 26 -50% patients<br>- 51 -75% patients<br>- 75% patients	9 (56.3)<br>6 (37.5)<br>1 (6.25)<br>0 (0)	12 (75.0)<br>2 (12.5)<br>1 (6.3)<br>1 (6.3)	0.33

**How do you describe the pre-transplant workup in ACLF patients in ICU, as compared with other situations?**			
- Similar to other situations	1 (6.2)	0 (0)	0.52
- Abbreviated workup to allow for a quick listing assuming that it will still be able to identify definitive contraindications to LT	11 (68.8	13 (81.3)	
- Abbreviated workup to allow for a quick listing with the risk of not identifying definitive contraindications to LT	4 (25.0)	3 (18.8)	
- More comprehensive workup to secure LT results in this very sick population	0 (0)	0 (0)	

Abbreviations : ACLF: Acute On Chronic Liver Failure, AUD: alcohol use disorder, AUDIT: Alcohol Use Disorders Identification Test, CCAS: coronary artery calcium scan, CCTA: coronary CT angiography, DSM: Diagnostic and Statistical Manual of Mental Disorders, ECG: electrocardiogram, EGD: oesophagograstroduodenoscopy, ENT: Ear-Nose-Throat, HRAR: High-Risk Alcoholism Relapse, Hosp: hospitalized patients without ALCF; LFI: liver frailty index, LT: liver transplantation, OutPat: outpatients, PSA: Prostatic Specific Antigen, RFH-NPT: Royal Free Hospital nutritional prioriziting tool, SALT: Sustained Alcohol Use After Early Liver Transplant, SGA: subjective global assessment, TAP: thoraco-abdomino-pelvic, TTE: trans-thoracic echocardiography

Table S4: Answers to the questionnaire regarding pre-transplant evaluation in ACLF-ICU patients among respondents from 37 French centers according to the center characteristic (liver transplant vs. non liver transplant center).

Question	French transplant centers	French non transplant centers	p value
**Cardiac and respiratory assessment**			
**Do you systematically perform an ECG?**			
Yes	16 (100)	20 (95.3)	0.38
No	0 (0)	1 (4.7)	
**Do you systematically perform a TTE?**			
Yes	16 (100)	21 (100)	1
No	0 (0)	0 (0)	
**Do you systematically request a stress exam?**			
Yes	0 (0)	1 (4.7)	0.38
No	16 (100)	20 (95.3)	
**Do you perform a stress exam in case of cardiovascular risk factors?**			
Yes	0 (0)	1 (4.7)	0.38
No	16 (100)	20 (95.3)	
**Do you systematically perform a CCTA or a CACS?**			
Yes	0 (0)	0 (0)	1
No	16 (100)	21 (100)	
**Do you perform a CCTA or a CACS in case of cardiovascular risk factors?**			
Yes	0 (0)	0 (0)	1
No	16 (100)	21 (100)	
**When do you perform a coronary angiography?**			
- In case of cardiovascular risk factors	4 (25.0)	4 (19.1)	0.66
- In case of symptoms	10 (62.5)	17 (81.0)	0.21
- In case of abnormal noninvasive tests	4 (25.0)	7 (33.3)	0.58
**What percentage of patients undergo a right-heart catheterism?**			
- < 25% patients	15 (93.8)	20 (95.2)	0.35
- > 25% of patients	1 (6.2)	1 (4.8)	

**When do you perform a right-heart catheterism in your center?**			
- Always	0 (0)	1 (4.8)	0.38
- In case of abnormal TTE results	15 (93.8)	20 (95.2)	0.84
- In case of symptoms	2 (12.5)	6 (28.6)	0.24
**When do you perform a doppler of supra-aortic trunks?**			
- Always	2 (12.5)	5 (23.8)	
- In case of risk factors	4 (25.0)	8 (38.1)	0.33
- Never	10 (62.5)	8 (38.1)	
**When do you request a cardiologist consultation?**			
- Always	6 (37.5)	6 (28.6)	
- In case of risk factors	3 (18.8)	6 (28.6)	0.75
- Never	7 (43.7)	9 (42.8)	
**What respiratory exam(s) do you systematically request?**			
- Thoracic CT scan	16 (100)	21 (100)	1
- Respiratory function test	2 (12.5)	8 (38.1)	0.08
- 6 minutes' walk test	0 (0)	0 (0)	1
- pneumologist consultation	1 (6.3)	1 (4.8)	0.84
**Addictology assessment**			
**How many patients are assessed by a senior addictologist?**			
- < 25% patients	2 (12.5)	6 (28.6)	
- 26-50% patients	2 (12.5)	2 (9.5)	
- 51-75% patients	5 (31.3)	1 (4.8)	0.14
- > 75% patients	7 (43.7)	12 (57.1)	
**If the first addictology assessment was realized in a nontransplant center, is it systematically performed again in the transplantation center?**			
- Yes	8 (50.0)	13 (61.9)	0.47
- No	8 (50.0)	8 (38.1))	
**Apart from alcohol-associated hepatitis, what is the minimal required duration of alcohol cessation to consider liver transplantation?**			

- ≥ 6 months<br>- 3-6 months<br>- 1-3 months<br>- < 1 month<br>- Case by case decision	1 (6.3)<br>0 (0)<br>2 (12.5)<br>0 (0)<br>13 (81.2)	1 (4.8)<br>8 (38.1)<br>0 (0)<br>0 (0)<br>12 (57.1)	 0.02
**Decisive components to predict alcohol relapse risk after transplantation?**			
**(1- most important, 8- less important)**			
- Addiction diagnosis according to DSM V	3 (1-8)	8 (2-8)	0.19
- Former failure of withdrawal cure	6 (5-8)	3 (1-6)	0.004
- Polyaddiction	6 (3-7)	4 (2-7)	0.39
- Psychiatric comorbidities	4 (3-7)	5 (3-6)	0.97
- Socio-professional insertion	4 (3-5)	4 (4-6)	0.28
- Relatives' quality	5 (4-6)	4 (3-6)	0.79
- Insight of their alcohol use disorder	4 (1-7)	3 (2-6)	0.96
- Addictologist follow-up	5 (2-7)	6 (5-7)	0.08
- **Which score(s) do you routinely use for your addictology assessment?**			
- AUDIT score	14 (87.5)	19 (90.5)	0.77
- HRAR score	3 (18.8)	3 (14.3)	0.72
- SALT score	1 (6.3)	2 (9.5)	0.72
**In which circumstances do you support your addictology assessment by questioning the relatives?**			
- Always	15 (93.8)	13 (61.9)	0.03
- In case of patient's encephalopathy/intubation	1 (6.2)	9 (42.9)	0.01
**Who is questioned in the family when this is done?**			
- The partner	16 (100)	20 (95.2)	0.38
- Children and parents	16 (100)	20 (95.2)	0.38
- Friends /colleagues	4 (25.0)	2 (9.5)	0.21
- The general practicioner	13 (83.8)	18 (85.7)	0.72
**How often do you perform blood, urinary or capillary screening tests?**			
- < 25% patients	9 (56.3)	13 (61.9)	
- 26 -50% patients	3 (18.7)	1 (4.8)	
- 51 -75% patients	0 (0)	1 (4.8)	0.48

- > 75% patients	4 (25.0)	6 (28.6)	
**Which biological or urinary test is most often performed in your center?**<br>- Carboxydate Deficience Transferrine<br>- Phosphatydyléthanol<br>- Urinary ethyl glucuronide<br>- Capillary ethyl glucoronide	 <br>5 (31.2)<br>6 (37.5)<br>4 (25.0)<br>1 (6.3)	 <br>7 (33.3)<br>10 (47.6)<br>3 (14.3)<br>1 (4.8)	 <br><br><br>0.84
**Do you always take into account the addictologist's opinion?**<br>- Yes<br>- No	 <br>12 (75.0)<br>4 (25.0)	 <br>15 (71.4)<br>6 (28.6)	 <br>0.81
**Oncologic assessment**<br>**In patients above 50 years old, what exams do you perform?**			
**When do you perform a colonoscopy**<br>- In all patients<br>- in case of risk factors of colorectal neoplasm	 1 (6.3)<br>4 (25.0)	 6 (28.6)<br>5 (23.8)	 0.09<br>0.93
**Do you systematically perform an upper GI endoscopy?**<br>- Yes<br>- No	 <br>11 (68.8)<br>5 (31.2)	 <br>16 (76.2)<br>5 (23.8)	 <br>0.61
**Do you systematically perform a CT scan of the abdomen and pelvis?**<br>- Yes<br>- No	 <br>11 (100)<br>0 (0)	 <br>21 (100)<br>0 (0)	 <br>1
**Do you systematically perform a PSA dosage in men?**<br>- Yes<br>- No	 <br>11 (68.8)<br>5 (31.2)	 <br>16 (76.2)<br>5 (23.8)	 <br>0.61
**Do you systematically perform a mammography, pap smear, and gynecologic exam for women?**<br>- Yes<br>- No	 <br>5 (31.2)<br>11 (68.8)	 <br>7 (33.3)<br>14 (66.7)	 <br>0.89
**Do you systematically perform an ENT consultation?**<br>- Yes	 <br>6 (37.5)	 <br>10 (47.6)	 <br>0.54

- No	10 (62.5)	11 (52.4))	
**Do you systematically perform a dermatology consultation?**<br>- Yes<br>- No	 <br>3 (18.8)<br>13 (81.2)	 <br>5 (23.8)<br>16 (76.2)	 <br>0.71
**Nutritional assessment**			
**When do you request nutritionist or dietitian consultation?**<br>- Systematically<br>- In case of clinical malnutrition<br>- Never	 <br>7 (43.7)<br>5 (31.3)<br>4 (25.0)	 <br>8 (38.1)<br>6 (28.6)<br>7 (33.3)	 <br><br>0.86
**When do you perform a screening for malnutrition using questionnaires such as SGA, RFH-NPT**<br>- Systematically<br>- In case of clinical malnutrition	 <br>2 (12.5)<br>1 (6.3)	 <br>6 (28.6)<br>4 (19.1)	 <br>0.24<br>0.26
**When do you perform a screening for sarcopenia using psoas measurement by CT scan?**<br>- Systematically<br>- In case of clinical malnutrition	 <br>3 (18.8)<br>3 (18.8)	 <br>0 (0)<br>3 (14.3)	 <br>0.04<br>0.71
**When do you perform a screening for frailty using dedicated tests (for example LFI)**?<br>- Systematically<br>- In case of clinical malnutrition	 <br>3 (18.8)<br>3 (18.8)	 <br>2 (9.5)<br>1 (4.8)	 <br>0.42<br>0.17
**General considerations**			
**In your experience, what is the timeframe in days between initiation of LT assessment and listing?**	5 (3-7)	10 (7-15)	<0.0001
**What proportion of ACLF patients develop ACLF while already listed for LT, with a complete pre-transplant workup?**<br>- < 25% patients<br>- 26 -50% patients<br>- 51 -75% patients<br>- 75% patients	 <br><br>9 (56.3)<br>6 (37.5)<br>1 (6.25)<br>0 (0)	 <br><br>17 (81.0)<br>3 (14.3)<br>1 (4.7)<br>0 (0)	 <br><br><br>0.24

**How do you describe the pre-transplant workup in ACLF patients in ICU, as compared with other situations?**<br>- Similar to other situations<br>- Abbreviated workup to allow for a quick listing assuming that it will still be able to identify definitive contraindications to LT<br>- Abbreviated workup to allow for a quick listing with the risk of not identifying definitive contraindications to LT<br>- More comprehensive workup to secure LT results in this very sick population	 <br><br>1 (6.2)<br>11 (68.8<br><br><br>4 (25.0)<br><br><br>0 (0)	 <br><br>0 (0)<br>15 (71.4)<br><br><br>5 (23.8)<br><br><br>1 (4.8)	 <br><br>0.55

Abbreviations : ACLF: Acute On Chronic Liver Failure, AUD: alcohol use disorder, AUDIT: Alcohol Use Disorders Identification Test, CCAS: coronary artery calcium scan, CCTA: coronary CT angiography, DSM: Diagnostic and Statistical Manual of Mental Disorders, ECG: electrocardiogram, EGD: oesophagograstroduodenoscopy, ENT: Ear-Nose-Throat HRAR: High-Risk Alcoholism Relapse, Hosp: hospitalized patients without ALCF; LFI: liver frailty index, LT: liver transplantation, OutPat: outpatients, PSA: Prostatic Specific Antigen, RFH-NPT: Royal Free Hospital nutritional prioriziting tool, SALT: Sustained Alcohol Use After Early Liver Transplant, SGA: subjective global assessment, TAP: thoraco-abdomino-pelvic, TTE: trans-thoracic echocardiography
