# Supplementary material for: Pre-liver transplant assessment of patients with acute-on-chronic liver failure: An international survey
Source: JHEP Rep. 2026 Feb 26;8(8):101794. doi: 10.1016/j.jhepr.2026.101794 (PMC13352056; doi:10.1016/j.jhepr.2026.101794)
Supplement: Multimedia component 4 [file mmc4.pdf]

Pre-liver transplant assessment of patients with acute-on-chronic liver failure: An international survey

Authors

Claire Bellec, Anea Augé, Sébastien L'Hermite, …, Alberto Zanetto, Laure Elkrief, **Florent Artru,**

Correspondence

l.elkrief@chu-tours.fr (L. Elkrief), florent.artru@univ-rennes.fr (F. Artru).

Graphical abstract

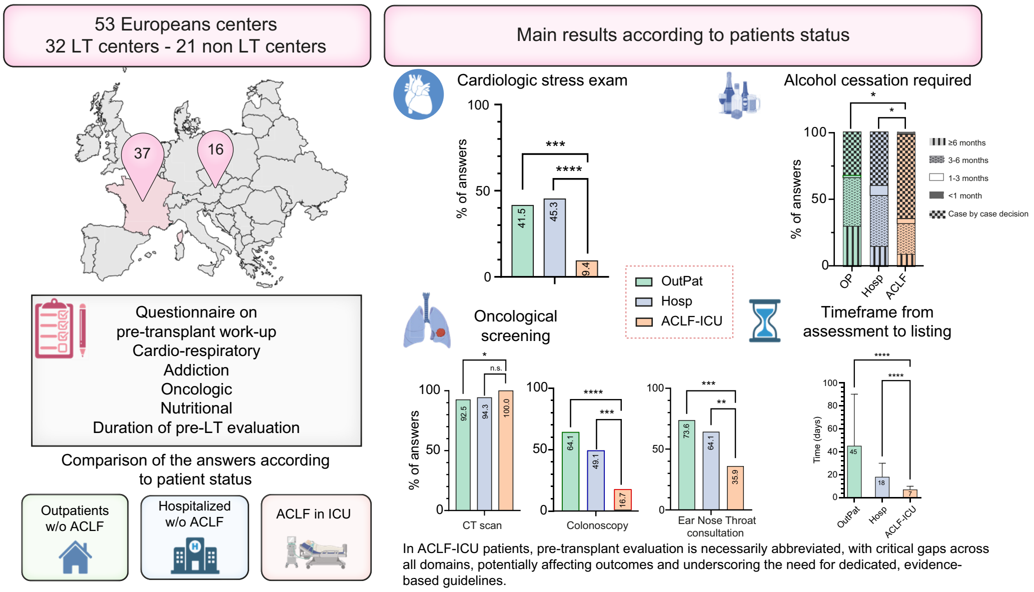

Highlights:

- Patients with ACLF admitted to the ICU undergo shorter pre-transplant evaluations than other candidates.
- Cardiological stress testing, coronary angiography, and specialist consultations are less frequently performed in ACLF-ICU patients.
- Oncological and nutritional assessments are markedly reduced during ICU-based transplant evaluation.
- Current pre-transplant evaluation strategies in ACLF rely largely on expedited, case-by-case decision-making.
- Patients listed after ACLF onset tend to experience more post-transplant cardiovascular events than those listed earlier.

Impact and implications:

We sent a questionnaire to centers with an expertise in the management of patients with ACLF to assess current practices in pre-transplant evaluation in patients with ACLF admitted to an intensive care unit, as compared with patients without (outpatients and patients hospitalized in a regular ward). According to the responses from 53 centers, cardiological stress test and coronary angiography were less commonly performed as part of the pre-transplant evaluation in patients with ACLF admitted to the ICU, as well as colonoscopy and ear, nose, and throat consultation. Median timeframe from pre-LT workup initiation to listing was 7 days in patients with ACLF admitted to the ICU, which was significantly shorter than in patients without ACLF. These results suggest that pre-transplant workup is abbreviated in patients with ACLF, and might have an impact on post-transplant outcome, especially cardiovascular complications. Further dedicated studies are needed to specifically address the relation between pre-transplant workup and post-LT complications in patients with ACLF.

https://doi.org/10.1016/j.jhepr.2026.101794
© 2026 The Author(s). Published by Elsevier B.V. on behalf of European Association for the Study of the Liver (EASL). This is an open access article under the CC BY license (http://creativecommons.org/licenses/by/4.0/).

Pre-liver transplant assessment of patients with acute-on-chronic liver failure: An international survey

Claire Bellec[1,2], Anea Augé[1], Sébastien L'Hermite[2], Romain Moirand[2], Armand Abergel[3], Matthew Amstrong[4], Teresa Antonini[5], Rodolphe Anty[6], Marion Khaldi[7], Aurore Baron[8], Mouni Bensenane-Oussalah[9], William Bernal[10], Christophe Bureau[11], Nicolas Carbonell[12], Jean-François Cadranel[13], Filipe Cardoso[14], Paul Carrier[15], Audrey Coilly[16], Isabel Conde[17], Filomena Conti[18], Agnes Bonadona[19], Gonzalo Crespo[20], Sarwa Darwish Murad[21], Sébastien Dharancy[22], Christophe Duvoux[23], Cornelius Engelmann[24], François Faitot[25], Claire Francoz[26], Armand Garioud[27], René Gerolami[28], Elia Gigante[29], Odile Goria[30], Thierry Gustot[31], Brian Hogan[32], Ludovic Lagin[33], Adrien Lannes[34], Marianne Latournerie[35], Victor de Ledinghen[36], Giulia Magini[37], Martin Mateo[38], Arnaud Maurin[39], Manuela Merli[40], Magdalena Meszaros[41], Georges-Philippe Pageaux[41], Claire Perignon[42], Giovanni Perricone[43], Michael Praktiknjo[44], Noemi Reboux[45], Thomas Reiberger[46], Valentin Rolle[47], Isabelle Rosa[48], Ruxandra Sarba[49], Faustine Wartel[50], Delphine Verhoeven Weil[51], Alberto Zanetto[52], **Laure Elkrief**[1,*,†], **Florent Artru**[2,*,†]

JHEP Reports **2026**. vol. 8 | 1–10

Check for updates

Background & Aims: Liver transplantation (LT) is increasingly performed in the setting of acute-on-chronic liver failure (ACLF). In this context, pre-transplant evaluation must be completed rapidly while minimizing the risk of overlooking contraindications. We aimed to describe current practices for pre-transplant assessment in patients with ACLF.

Methods: We conducted a survey across 34 European LT centers (including 16 French) and 22 French non-LT centers to assess pre-transplant evaluation practices, focusing on cardiopulmonary, addiction, oncological, and nutritional assessments. Practices were compared across three clinical scenarios: outpatients (OutPat), hospitalized decompensated patients without ACLF (Hosp), and patients with ACLF admitted to intensive care units (ACLF-ICU). In parallel, we retrospectively evaluated post-transplant outcomes in patients with cirrhosis and severe ACLF transplanted in two high-volume centers.

Results: Fifty-three centers (96%) responded. Cardiological stress testing was reported in 2% of ACLF-ICU patients *vs.* 21% of OutPat (p = 0.002) *vs.* 17% of Hosp patients (p = 0.008). Coronary angiography following abnormal non-invasive testing was less frequently performed in ACLF (42%) than in OutPat (76%, p = 0.0004) or Hosp patients (66%, p = 0.01). Alcohol abstinence requirements were more often decided on a case-by-case basis in patients with ACLF (62%) than in OutPat or Hosp patients. Oncological screening, including colonoscopy and ear, nose, and throat consultation, was also less frequently performed in ACLF-ICU patients. Median time from assessment initiation to listing was 7 days in ACLF-ICU *vs.* 45 days in OutPat and 18 days in Hosp patients (both p <0.0001). In the retrospective cohort (n = 221), patients listed after ACLF onset had a higher 1-year incidence of cardiovascular events than those listed before ACLF onset (19% *vs.* 9%).

Conclusions: In ACLF-ICU patients, pre-transplant evaluation is markedly abbreviated, with critical gaps—particularly in cardiological assessment—highlighting the need for dedicated, evidence-based guidelines.

© 2026 The Author(s). Published by Elsevier B.V. on behalf of European Association for the Study of the Liver (EASL). This is an open access article under the CC BY license (http://creativecommons.org/licenses/by/4.0/).

Introduction

Liver transplantation (LT) is a saving-life treatment for patients with cirrhosis and end-stage liver disease, or hepatocellular carcinoma within transplant criteria. Furthermore, 10- and 20-year post-transplant survival is around 60% and 50%, respectively.[1–3] Thus, LT is now regarded to be a durable therapy of choice for a broad range of conditions that severely impair hepatic function. Acute-on-chronic liver failure (ACLF) is a common and severe clinical syndrome, defined by the acute development of extrahepatic organ failures requiring hospitalization in an intensive care unit (ICU) and is associated with a high risk of short-term mortality.[4] In the absence of specific therapies, LT remains the only curative option.[4] It is particularly indicated in patients with persistent (*i.e.* after few days of intensive care management) severe ACLF who exhibit the highest risk of death in the absence of spontaneous recovery.[5] Growing evidence supports the feasibility of bridging carefully selected patients with multi-organ failure to LT, with favorable outcomes observed both in the short- and long-term.[5,6]

* Corresponding authors. Addresses: CHRU de Tours et Faculté de Médecine de Tours, Service d'Hépato-Gastroentérologie, Hôpital Trousseau, Avenue de la République, 37170 Chambray-lès-Tours, France. Tel.: +33 2 4747 5965 (L. Elkrief); Liver Department, Rennes University Hospital and Rennes University, 2 rue Henri le Guilloux, Rennes, France. Tel.: +33 2 9928 43 21. (F. Artru).
E-mail addresses: l.elkrief@chu-tours.fr (L. Elkrief), florent.artru@univ-rennes.fr (F. Artru).
† Shared last authors.
https://doi.org/10.1016/j.jhepr.2026.101794

A comprehensive, multidisciplinary pre-transplant evaluation is key to maintain good post-LT outcome. Particular attention is paid to the screening for the most common and severe comorbidities that negatively impact short- and long-term post-LT outcomes, namely cardiovascular risk, *de novo* extrahepatic malignancies, high risk of alcohol relapse, or frailty.[7–11] Despite their younger age, such comorbidities are also common in patients with ACLF evaluated for LT.[12–14]

The importance of a rigorous pre-transplant assessment has been emphasized by major transplant societies, including EASL, AASLD, and Asia-Pacific Association for the Study of the Liver (APASL) which have proposed structured frameworks to guide clinicians.[8,15,16] In non-urgent patients, namely either outpatients or patients hospitalized in a regular ward, the duration of the pre-transplant workup directly impacts post-LT outcomes as an early evaluation within 30 days after first assessment was shown to be associated with a lower pre-LT mortality.[17] Optimizing this timeframe appears even more critical in patients with ACLF considering the natural history of the syndrome. Although some LT candidates develop ACLF after being listed, the majority undergo their pre-transplant evaluation during an ACLF episode following ICU admission.[5,12,13] Hence, in this situation, the pre-LT evaluation must be completed rapidly to reduce mortality risk before placement on the waiting list (WL) and while listed to optimize access to LT.

To what extent the pre-LT evaluation is adapted in the context of patients with ACLF hospitalized in ICU remains largely unknown.

Specifically, it is unclear which investigations need to be conducted in ACLF-ICU patients evaluated for LT. These patients require continuous monitoring, are often bedridden, have altered mental status, and receive organ support such as mechanical ventilation, vasopressors, or renal replacement therapy. Key aspects such as the feasibility of functional cardiopulmonary assessment, oncological screening and the evaluation of psychosocial and addiction-related factors in this setting have not been described. Addressing these knowledge gaps is essential to promote the harmonization of clinical practices, with the dual objective of improving the detection of contraindications to LT and ensuring timely access to transplantation with optimal post-transplant outcomes.

In the present study, we aimed to characterize how the pre-LT workup is conducted in patients with ACLF admitted in ICU, in comparison with outpatients and hospitalized individuals with decompensated advanced chronic liver disease. For this purpose, we conducted a survey of current practices across European LT centers and non-transplant centers (in France). In parallel, we further aimed to investigate, in two high-volume LT centers with extensive experience in transplanting patients with severe ACLF, how pre-LT evaluations were practically conducted, and whether they were associated with post-LT outcomes.

Patients and methods

Survey

We designed a declarative survey to assess current practices in the pre-LT evaluation of patients with decompensated advanced chronic liver disease across French and European centers, including both LT and non- LT centers. All non-LT centers were French. They were selected by the study co-ordinators based on their expertise in the care of patients with liver diseases, including patients with ACLF. The survey focused on three patient populations commonly considered for LT: outpatients (OutPat), hospitalized patients in conventional wards without ACLF (Hosp), and patients with ACLF admitted to intensive care units (ACLF-ICU). The questionnaire was developed by two LT hepatologists (FA and LE) and one addiction specialist (RM), all affiliated with LT centers.

Briefly, the questionnaire was structured into three parts corresponding to the patient status at the time of pre-transplant evaluation (OutPat, Hosp, and ACLF), each encompassing four thematic domains, and general questions.

Cardiopulmonary assessment

This section included 12 questions covering routine investigations (*e.g.* electrocardiogram [ECG], transthoracic echocardiography [TTE], pulmonary function tests, chest computed tomography [CT] scan), their specific indications (*e.g.* coronary angiography, right heart catheterization), and referral to cardiologists and/or pulmonologists. The content was identical across the three parts.

Addiction assessment

This section included 10 questions addressing: the proportion of patients assessed by a senior addiction specialist, the systematic nature of addiction evaluation in transplant centers, the minimum duration of abstinence required (excluding alcohol-associated hepatitis), perceived relapse risk factors, tools or scores used, assessment of social support, use of biological markers (blood or urine), and the estimated proportion of patients deemed unsuitable for LT because of addiction. The content was identical across the three parts.

Oncological assessment

This section consisted of seven questions on whether specific cancer screening tests were performed (*e.g.* mammography, thoracoabdominal-pelvic CT, upper gastrointestinal (GI) endoscopy, dermatology consultation) in patients >50 years old. The content was identical across the three parts.

Nutritional assessment

This section included three questions on whether patients were assessed by a dietician or nutritionist, and which tools were used to screen for malnutrition, sarcopenia, and frailty (clinical, imaging-based, or dedicated tests).

General questions

In addition, we recorded the declared number of evaluated and/or transplanted patients per year in each center. At the end of each part, participants were asked to estimate the average time (in days) between the beginning of the pre-LT assessment and activation on the WL (free-text response). Finally, respondents were asked to provide a subjective evaluation of the quality of the pre-LT assessment in patients with ACLF patients.

Survey administration

The questionnaire was distributed via Google Forms. All questions were mandatory. The questionnaire included single-choice, multiple-choice, and open-ended items. The survey was sent to 38 French centers, including the 16 LT centers and 22 non-transplant centers (15 university hospitals and seven general hospitals with a high expertise in management of patients with cirrhosis, including patients with ACLF), as well as to 18 large-volume European LT centers. One to three hepatologists were contacted in each center; however, they were asked to provide only one single, consensus-based response per center. Each completed questionnaire was therefore intended to reflect the collective practices and attitudes within that specific institution.

Objectives

The main objective was to evaluate pre-transplant assessment in ACLF-ICU patients, as compared with patients either followed in outpatient clinic or admitted to a regular ward at the time of pre-LT assessment. We thus compared answers to the questionnaire according to patient' status at the time of pre-LT evaluation (ACLF *vs.* other).

The secondary objective was to evaluate disparities across centers. We thus compared pre-transplant evaluation in ACLF-ICU patients between (i) French LT centers and other European LT centers and (ii) French LT centers and French non-LT centers.

Retrospective study on pre-LT evaluations

We included all patients who underwent LT for severe ACLF (grade 2 or 3 according to EASL-CLIF [Chronic Liver Failure Consortium] criteria) between January 1, 2014, and December 31, 2023, at the University Hospitals of Rennes and Tours. We excluded patients <18 years, those who underwent multi-organ transplantation, and those with a history of prior LT.

We retrospectively collected demographic characteristics, etiology of underlying liver disease, ACLF grade at the time of LT, date of placement on the WL and whether listing occurred before or after ACLF onset, modalities of cardiological and oncological evaluations performed before WL listing, and the time interval between WL placement and transplantation.

Post-transplant outcomes included 1-year overall survival, the occurrence and timing of major adverse cardiovascular events (MACE), defined as a composite of cardiovascular death, myocardial infarction, stroke or transient ischemic attack, hospitalization for acute heart failure, or life-threatening ventricular arrhythmia, as well as the diagnosis of *de novo* malignancy at any site, including dermatological cancers, within 1 year after transplantation.

This study was conducted in accordance with the Declaration of Helsinki. According to French regulations, this retrospective observational study using routinely collected, anonymized data was outside the scope of the Jardé law, the requirement for informed consent was waived. The study was approved by our institutional review board, Tours University Hospital Ethics Committee in Human Research (number 2025 051).

Statistical analysis

Quantitative variables are presented as medians with IQR, and qualitative variables as absolute counts with corresponding percentages. Comparisons across patient status in the survey (outpatients [OutPat], hospitalized patients in conventional wards without ACLF [Hosp], and patients with ACLF in intensive care units [ACLF-ICU]) or in the retrospective study (placed on WL before or after ACLF onset) were performed using the Mann–Whitney U test for quantitative variables, and Pearson's X^2 test or Fisher's exact test, as appropriate, for qualitative variables. Single-choice questions were analyzed as categorical variables using global response distributions. Multiple-choice questions were analyzed on a per-item basis, with each response option treated as a binary variable (selected *vs.* not selected). Open-ended responses were analyzed using a qualitative descriptive approach to identify recurring themes and patterns.

A two-sided p value of <0.05 was considered statistically significant. All analyses were performed using NCSS version 2024 (NCSS [Number Cruncher Statistical System], LLC, Kaysville, UT, USA) and GraphPad Prism version 11.0 (GraphPad Software, San Diego, CA, USA).

Results

Results of the survey

Of the 55 invited centers, 53 (96.4%) responded to the survey. This included 37/38 (97.4%) French centers (16/16 LT centers and 21/22 non-LT centers) and 16/18 (88.9%) European LT centers (from eight countries, including Austria, Belgium, Germany, Italy, Portugal, Spain, Switzerland, and UK). The list of the centers is shown in Table S1.

Comparison of pre-transplant assessment in ACLF-ICU patients compared with OutPat and Hosp patients

Among the 16 French transplant centers, six centers (38 %) reported performing >100 LTs annually, six centers (38%) performed between 51 and 100 LTs, three centers (19%) between 21 and 50 LTs, and one center (6%) <20 LTs per year. Regarding LT specifically in the setting of ACLF, three transplant centers (19%) reported conducting less than five LTs annually, three (19%) between six and 10, five (31%) between 11 and 20, and five (31%) >20 ACLF-related LTs per year. Among the 16 European transplant centers, seven centers (44%) reported performing more than 100 LTs annually, four centers (25%) performed between 51 and 100 LTs, and five centers (31%) between 21 and 50 LTs per year. Regarding LT specifically in the setting of ACLF, five transplant centers (31%) reported conducting less than five LTs annually, 10 (63%), and one (6%) >20 ACLF-related LTs per year.

In transplant centers, there was an association between the report of annual total number of LTs performed and the declared number of LTs performed in the context of ACLF (p = 0.01). For instance, four of the five centers performing >20 ACLF-related LTs per year also reported >100 LTs annually.

Among the 21 non-transplant centers, referral volumes to LT centers for LT evaluation varied: 11 centers (52%) referred one to 20 patients per year, seven (33%) referred 21–50, and three (14 %) referred >50 patients annually. Specifically in the context of ACLF, 10 non-transplant centers (48%) reported

referring one to five patients per year, nine (43%) referred six to 10 patients, and two (10%) referred >10 patients annually for LT evaluation.

Table S2 summarizes all results from the comparison of answers from the 53 centers according to patient's setting at the time of pre-transplant evaluation.

Regarding cardiac assessment, although ECG and TTE were almost systematically reported to be performed across all groups, stress testing was reported to be conducted in 2% of ACLF-ICU patients, *vs.* 21% in OutPat (p = 0.002) and 17% in Hosp (p = 0.008) patients. In the same line, coronary angiography was reported to be performed in case of abnormal non-invasive tests in only 42% in ACLF-ICU, *vs.* in 76% in OutPat (p = 0.0004) and 66% in Hosp (p = 0.01). Coronary artery calcium scan (CACS) and coronary CT angiography (CCTA) were reported to be performed in case of cardiovascular risk factors in 4% of centers for ACLF-ICU *vs*. 21% in OutPat (p = 0.008) and 21% in Hosp (p = 0.008). Notably, supra-aortic Doppler ultrasound as well as cardiologists' consultation were reported to be less frequent in ACLF-ICU patients than in other groups (Fig. 1).

Regarding respiratory assessments, although thoracic CT scans were reported to be performed almost systematically in all groups, pulmonary function tests were also reported to be less commonly performed in ACLF-ICU (25%) than either in OutPat (89%, p <0.0001) or in Hosp (76% p <0.0001) patients. Similarly, a pneumologist consultation was reported in only 6% of ACLF-ICU, *vs*. in 19% in OutPat (p = 0.04) and 15% in Hosp (p = 0.1) (Fig. 2).

In terms of addiction evaluation, the reported minimal required duration of alcohol abstinence before LT varied across patient groups, with a significantly higher reliance on case-by-case decision-making in the ACLF-ICU group (62%) compared with OutPat (32%, p = 0.008) and Hosp (40%, p <0.0001) (Fig. 3A). None of the proposed decisive components to predict alcohol relapse risk after transplantation significantly differed across groups (Fig. 3B). The Alcohol Use Disorders Identification Test (AUDIT) score was the primary tool used for routine addiction assessment, reported by >80% of respondents across all clinical scenarios. In contrast, the use of alcohol consumption biomarkers was more heterogeneous and evenly distributed among carbohydrate-deficient transferrin, phosphatidylethanol, and urinary ethyl glucuronide, with no significant differences across scenarios.

Regarding oncological assessment in patients older than 50 years, systematic colonoscopy was reported to be performed in 17% of ACLF-ICU patients *vs*. 64% and 49% in OutPat and Hosp patients, respectively (p <0.0001 and p = 0.0004, respectively). Mammography/gynecologic exams were reported to be performed in 30% in ACLF-ICU, *vs*. 89% and 70% in OP and Hosp, respectively (p <0.0001 and p = 0.001, respectively). Ear, nose, and throat (ENT) consultation (36% in ACLF-ICU *vs.* 74% in OutPat, p = 0.0001 and *vs.* 64% in Hosp, p = 0.004) and dermatology consultation (17% in ACLF-ICU *vs*. 47% in OutPat, p = 0.0009 and *vs*. 43% in Hosp, p = 0.004) were declared less frequently performed in ACLF-ICU patients (Fig. 4).

Regarding nutritional assessment, 36% of the centers reported never referring patients for nutritional consultation in ACLF-ICU patients (*vs.* 0% in OutPat, p <0.0001 and *vs*. 23% in Hosp, p = 0.003). Screening for frailty in case of clinical malnutrition was reported to be performed in 11% in ACLF-ICU patients (*vs*. 43% in OutPat, p = 0.0002 and *vs*. 45% in Hosp, p = 0.0001).

Finally, declared median time from assessment to listing varied markedly across groups: 45 days (30–90) for OutPat, *vs.* 18 days (14–30) for Hosp, and *vs.* 7 days (3–10) for ACLF-ICU patients (p <0.0001 for both comparisons) (Fig. 5). Seventy-four percent of the centers reported that the pre-LT evaluation in patients with ACLF patients is intentionally abbreviated to enable rapid listing, based on the assumption that absolute contraindications can still be identified. By contrast, 23% of the centers acknowledged that such an abbreviated assessment carries a risk of failing to detect absolute contraindications to transplantation.

Comparison of pre-transplant assessment in ACLF-ICU patients between French and European LT centers

Table S3 summarizes all results from the comparison of responses in the ACLF group between French and European LT centers.

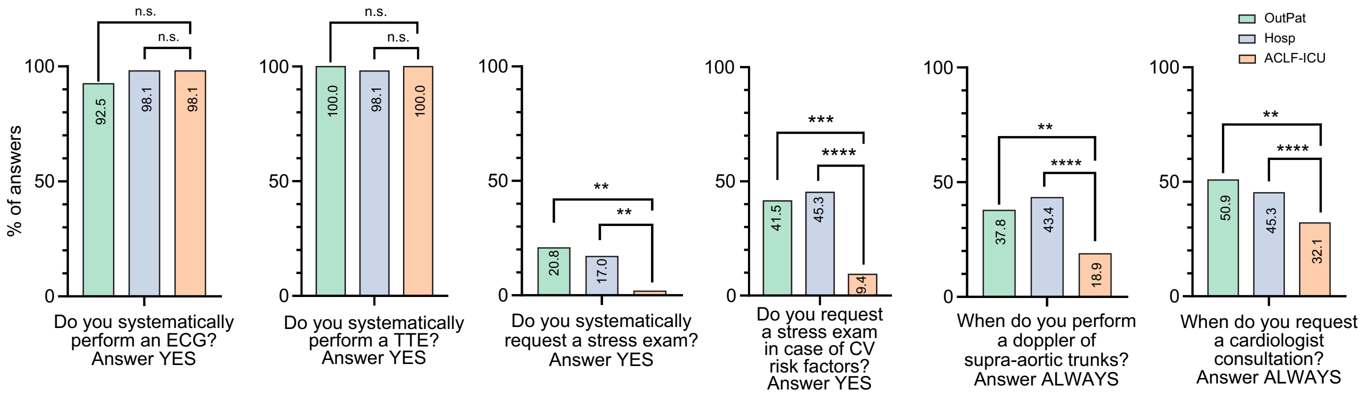

Fig. 1. Summary of answers regarding pre-liver transplantation cardiac assessment according to patient's setting in the 53 centers. Comparisons across patient status were performed using the Mann–Whitney *U* test for quantitative variables, and Pearson's X^2 test or Fisher's exact test, as appropriate, for qualitative variables. *p <0.05, **p <0.01, ***p <0.001, ****p <0.0001. ACLF, acute-on-chronic liver failure, ECG, electrocardiogram; Hosp, hospitalized patients; NS, non-significant; OutPat, outpatients, TTE, transthoracic echocardiography.

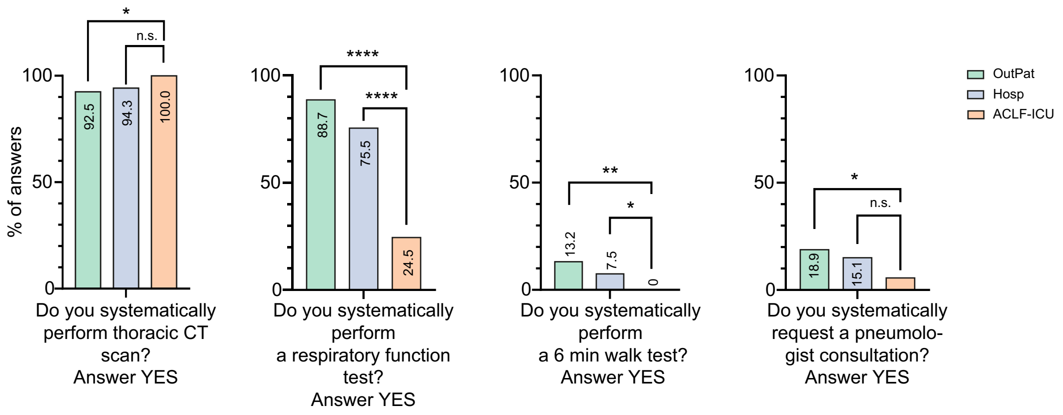

Fig. 2. Summary of answers regarding pre-liver transplantation respiratory assessment according to patient's setting in the 53 centers. Comparisons across patient status were performed using the Mann–Whitney *U* test for quantitative variables, and Pearson's X^2 test or Fisher's exact test, as appropriate, for qualitative variables. **p* <0.05, ***p* <0.01, ****p* <0.001, *****p* <0.0001. ACLF, acute-on-chronic liver failure; Hosp, hospitalized patients; NS, non-significant; OutPat, outpatients.

Regarding cardiac assessment, although ECG, TTE, and thoracic CT scan were uniformly performed across all centers, European centers declared to perform more frequently coronary angiography, in the case of abnormal non-invasive tests (69% *vs.* 25%, *p* = 0.01) while French centers more often relied on symptomatic presentation (63% *vs.* 25%, *p* = 0.03). European centers also declared more frequently referring patients to the cardiologist consultation in case of cardiovascular risk factors (63 *vs.* 19%, *p* = 0.02).

Regarding addiction evaluation, a significantly higher proportion of European centers reported systematically repeating the addiction assessment at the transplant center when the initial evaluation was performed outside the LT center (94% *vs.* 50%, *p* = 0.006). Furthermore, European centers reported to place less emphasis on relative interviews than French centers (63 % *vs*. 94%, *p* = 0.03), and less likely to report involving a general practitioner interview (38% *vs*. 84%, *p* = 0.01). A history of failed alcohol withdrawal cure was declared as considered more decisive for predicting alcohol relapse after LT (*p* = 0.0003), whereas socio-professional insertion (namely presence of relatives, and job) was declared as deemed less critical (*p* = 0.003) than in French centers. Oncologic and nutritional assessment, showed similar practices across regions, with both groups reporting low rates of systematic screenings for frailty, sarcopenia, and malnutrition using standardized tools. Although the primary tool used for routine addiction assessment did not differ between French and other European LT centers, differences were observed in the reported use of alcohol consumption biomarkers. Phosphatidylethanol was numerically more frequently used in French centers (37.5%) compared with other European centers (6.3%), whereas urinary ethyl glucuronide was numerically more frequently used in European centers; however, these differences did not reach statistical significance.

The time from initiation of assessment to listing was comparable, and a majority of both French and European centers reported relying on an abbreviated workup in ACLF-ICU patients to facilitate timely listing without risking of missing definitive contraindications.

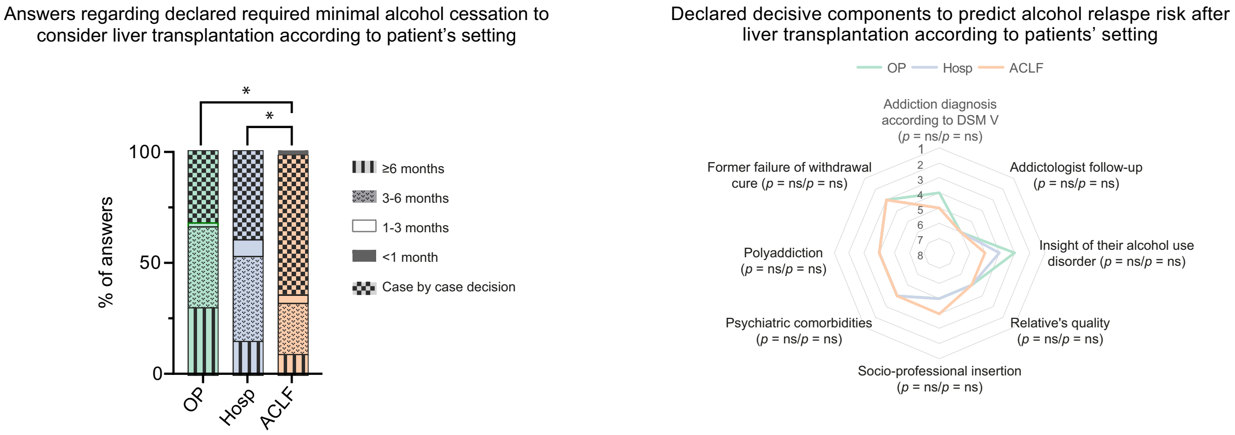

Fig. 3. Summary of answers regarding pre-liver transplantation addiction assessment according to patient's setting in the 53 centers. Comparisons across patient status were performed using the Mann–Whitney *U* test for quantitative variables, and Pearson's X^2 test or Fisher's exact test, as appropriate, for qualitative variables. **p* <0.05, ***p* <0.01, ****p* <0.001, *****p* <0.0001. ACLF, acute-on-chronic liver failure; DSM, *Diagnostic and Statistical Manual of Mental Disorders*; Hosp, hospitalized patients; NS, non-significant; OutPat, outpatients.

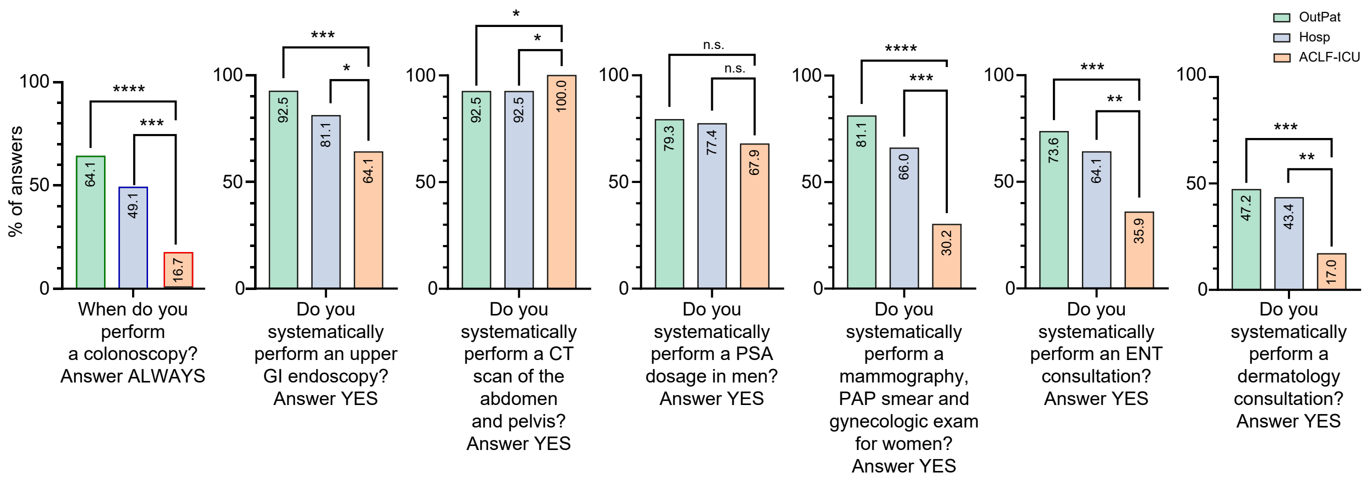

Fig. 4. Summary of answers regarding pre-liver transplantation oncological assessment in patients ≥50 years old in the 53 centers. Comparisons across patient status were performed using the Mann–Whitney *U* test for quantitative variables, and Pearson's X^2 test or Fisher's exact test, as appropriate, for qualitative variables. **p* <0.05, ***p* <0.01, ****p* <0.001, *****p* <0.0001. ACLF, acute-on-chronic liver failure, CT scan, cardiothoracic scan; GI endoscopy, gastrointestinal endoscopy; Hosp, hospitalized patients; NS, non-significant; OutPat, outpatients; ORL, Oto-rhino-laryngologist; PSA, prostatic specific antigen.

Notably, when comparing the 10 LT centers who reported to perform five or fewer ACLF-related LTs per year ('low-volume centers') to higher-volume centers, the only significant difference observed in pre-transplant assessment practices was cardiology involvement. Specifically, 80% of 'low-volume' centers reported routine evaluation by a cardiologist *vs.* only 14% among higher-volume centers (*p* = 0.001).

Comparison of pre-transplant assessment in ACLF-ICU patients between French LT and non-LT centers

Table S4 summarizes all results from the comparison of responses in the ACLF group between French LT and non-LT centers. Regarding the cardiopulmonary assessment, the answers to the questionnaire did not significantly differ between French LT centers and LT centers. Regarding addiction assessment, transplant centers were more likely to base transplant decisions on a case-by-case evaluation of alcohol abstinence than non-transplant centers (81% *vs.* 57%, *p* = 0.02), whereas non-transplant centers more frequently declared to adhere to defined periods of abstinence. Furthermore, family involvement in the addiction assessment was more systematic in transplant centers (94% *vs*. 62%, *p* = 0.03). A history of failed alcohol withdrawal was declared as considered more decisive for predicting alcohol relapse after LT in non-transplant centers (*p* = 0.004). Regarding oncologic and nutritional assessment, answers did not significantly differ between transplant and non-transplant centers. The declared timeframe between initiation of LT assessment and listing was declared to be shorter in transplant centers than in non-transplant centers (5 days *vs*. 10 days, *p* <0.0001).

Results of the retrospective study

A total of 221 patients underwent LT during the study period at the University Hospitals of Rennes and Tours. Their main baseline characteristics are summarized in Table 1. Briefly, 159 (71.6%) patients were male, with a median age of 57.2 (IQR 49–62.4) years, and the leading etiologies of underlying liver disease were alcohol, metabolic syndrome, hepatis B virus, and hepatitis C virus in 170 (76.6%), 47 (21.2%), 15 (6.8%), and 14 (6.3%) patients, respectively. The median model for end-stage liver disease (MELD) was 36 (IQR 31–41). Among these patients, 73 (33.0%) were placed on the WL before ACLF onset, whereas 148 (67.0%) were listed after ACLF onset. A comparison of pre-LT evaluations between these two groups is provided in Table 1.

Compared with patients already listed at the time of ACLF onset, those listed after ACLF onset experienced a significantly longer interval between WL placement and transplantation (median 49 days [IQR 18–210] *vs.* 4 days [IQR 2–9], *p* <0.0001). With regard to cardiological assessment, patients listed after ACLF onset underwent stress testing significantly less frequently (41.9% *vs.* 68.5%, *p* = 0.0002). Similarly, oncological evaluations were less frequently performed in patients

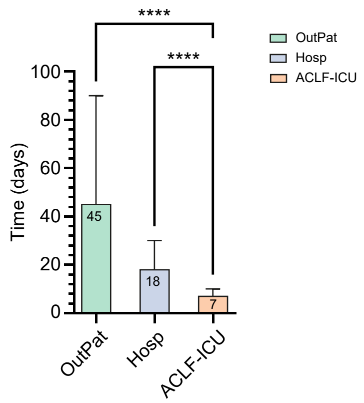

Fig. 5. Answers regarding declared timeframe between initiation of liver transplantation assessment and placement on the waiting list according to patient's setting in the 53 centers. Comparisons across patient status were performed using the Mann–Whitney *U* test for quantitative variables, and Pearson's X^2 test or Fisher's exact test, as appropriate, for qualitative variables. **p* <0.05, ***p* <0.01, ****p* <0.001, *****p* <0.0001. ACLF, acute-on-chronic liver failure; Hosp, hospitalized patients; NS, non-significant; OutPat, outpatients.

Table 1. Association between pre-LT workup and post-transplant outcomes in 221 patients with severe ACLF at LT in two large-volume centers.

	Placed on WL before ACLF onset (n = 73)	Placed on WL after ACLF onset (n = 148)	p values
ACLF grade at LT	2 (2–3)	2 (2–3)	0.86
Delay listing to LT, days	49 (18–120)	4 (2–9)	<0.0001
Cardiological evaluation			
TTE performed	69 (94.5)	142 (96.0)	0.63
Stress exam performed	50 (68.5)	62 (41.9)	0.0002
CCTA or CACS performed	4 (5.6)	4 (2.7)	0.29
Coronarography performed	9 (12.5)	9 (6.1)	0.10
Cardiologist consultation performed	26 (36.1)	38 (25.9)	0.11
Oncological evaluation			
CT scan abdomen and pelvis performed	71 (97.2)	141 (95.9)	0.29
Colonoscopy performed	22 (30.1)	24 (16.2)	0.01
Upper gastrointestinal endoscopy	57 (78.0)	110 (74.3)	0.33
ENT consultation performed	49 (67.1)	77 (52.0)	0.02
Mammography, pap smear, and gynecologic exam for women performed	8 (50.0)	16 (34.0)	0.25
Dermatology consultation performed	45 (61.6)	71 (47.9)	0.03
Post-transplant outcomes			
MACE at 1 year	7 (9.6)	28 (18.9)	0.07
De novo malignancies at 1 year	3 (4.1)	12 (8.1)	0.28

Data are presented as median (IQR) or n (%). Continuous variables were compared using the Mann–Whitney *U* test, and categorical variables using Pearson's X^2 or Fisher's exact test, as appropriate. ACLF, acute-on-chronic liver failure; CACS, coronary artery calcium scan, CCTA, coronary CT angiography; ENT, ear, nose and throat; LT, liver transplantation; MACE, major cardiovascular events; MELD, model for end-stage liver disease; TTE, transthoracic echocardiography.

listed after ACLF onset, including colonoscopy (16.2% *vs.* 30.1%, *p* = 0.01), otorhinolaryngology consultation (52.0% *vs.* 67.1%, *p* = 0.02), and dermatological examination (47.9% *vs.* 61.6%, *p* = 0.03).

At 1 year after transplantation, patients who were listed after ACLF onset had a numerically higher incidence of MACE compared with those listed before ACLF onset (18.9% *vs.* 9.6%), although this difference did not reach statistical significance (*p* = 0.07). Similarly, the incidence of de novo malignancies was numerically higher in patients listed after ACLF onset (8.1% *vs.* 4.1%), without a statistically significant difference between groups (*p* = 0.28).

Discussion

The present international survey provides novel and real-life insights into the pre-transplant assessment in patients with ACLF hospitalized in ICU. This survey is the first to explore how ACLF modifies pre-transplant evaluation across a large national and European network. The strength of the present questionnaire is that it involved 53 European centers from nine European countries.

The first major finding is that the pre-LT workup in patients with ACLF is substantially abbreviated compared with other candidates, such as outpatients (OutPat) or non-ACLF hospitalized (Hosp) patients. Regardless of the mechanism used to ensure rapid access to LT—whether based on MELD score alone, as in France, or through specific ACLF prioritization tiers, as implemented in the UK and Spain—respondents consistently reported shorter timeframes for pre-LT evaluations in these patients. Although such an approach may raise concerns regarding the potential under-detection of critical contraindications before transplantation, it is primarily adopted to allow rapid placement on the WL and thereby optimize timely access to LT. This is in line with recent guidelines, that recommend a rapid evaluation, given the high short-term mortality in ACLF.[8,18]

The second major finding of this survey is the limited scope of cardiovascular assessment in patients with ACLF evaluated for LT. Surprisingly, respondents declared a low rate of referral for formal cardiology consultation. This finding was somewhat unexpected and may be explained, at least in part, by the high level of expertise of the participating centers, which are accustomed to managing these complex clinical situations. In such settings, intensivists, transplant hepatologists, and anesthesiologists often jointly assess cardiovascular transplantability, allowing for rapid multidisciplinary decision-making without systematic cardiology referral. Although baseline investigations such as ECG and echocardiography are systematically performed, stress testing and invasive coronary angiography were reported to be rarely performed in this population. This practice diverges sharply from what is typically implemented in stable patients. It can be speculated that both medical, such as the risk of developing acute kidney injury, and logistic concerns (*e.g.* transport of unstable patients) may explain these discrepancies. However, accumulating evidence suggesting that cardiovascular mortality may be higher after LT in patients with ACLF.[6,19] Interestingly, we found that the incidence of MACE within 1 year after LT was higher in patients who were placed on the WL after onset of ACLF—namely those with an abbreviated pre-LT workup —than in those who were placed on the WL before onset of ACLF—namely those with a more extensive workup. Further larger studies are needed to validate these preliminary results and to identify patients with a high risk of cardiovascular complications after LT, and requiring a more extensive evaluation. Although currently poorly used, targeted approaches using CT-based imaging modalities (such as CACS and CCTA) and dedicated risk stratification tools such as the coronary artery disease-liver transplantation score (CAD-LT) score—independently associated with post-transplant cardiovascular events—could enhance cardiovascular risk assessment in ACLF candidates, particularly those with metabolic comorbidities or a history of cardiovascular disease.[20–23] Selective application of these non-invasive screening strategies may help identify high-risk patients who could benefit from focused invasive evaluation, even in the context of critical illness. Such a strategy may represent a pragmatic balance between rapid access to transplantation and patient safety, mitigating the risks associated with a de-escalation of cardiovascular assessment in ICU settings. As an example, performing coronary angiography only in candidates with a CAD-LT score ≥9—or preferably ≥11—may enhance its diagnostic yield, with significant coronary lesions being detected in ~40–60% of cases in this high-risk subgroup.[22,23] This selective approach may help rationalize the use of invasive testing, which remains feasible when indicated, particularly given the prognostic

implications of proceeding or deferring LT based on coronary status.[22–24]

Another major challenge highlighted in our survey is the reduced scope and reliability of addiction assessments in patients with ACLF. A large proportion of centers reported relying on expedited, case-by-case addiction assessments, primarily based on AUDIT scores and alcohol consumption biomarkers, with involvement of addiction specialists and interviews with relatives. This streamlined approach is understandable given the urgency of transplantation decisions in ICU settings, but it raises concerns, especially in the context of alcohol-associated liver disease. The declared case-by-case approach regarding the required duration of alcohol abstinence before transplantation may derive from emerging evidence in the field of alcohol-associated hepatitis.[25,26] However, in the prospective controlled QuickTrans trial, a stringent psychosocial selection process failed to demonstrate the non-inferiority of early LT (without a 6-month abstinence period) in terms of post-transplant alcohol relapse risk when compared to the standard strategy. Notably, the relapse rate was numerically higher in the early transplant group (34% *vs*. 25%), and high-risk alcohol consumption occurred significantly more frequently (22% *vs*. 5%), raising concerns about long-term behavioral outcomes despite rigorous pre-transplant evaluation. Currently, no data specifically address post-transplant relapse rates in patients transplanted for ACLF directly from the ICU. The present survey highlighted the difficulty of accurate addiction evaluation in ACLF-ICU patients, because of altered mental status and the short time allowed. Developing and validating predictive tools tailored to ICU patients could improve the balance between timely access to life-saving transplantation and post-transplant safety. Until then, enhancing the addiction assessment—even if abbreviated—through structured criteria, repeated assessments when possible, and early involvement of specialized teams before and after transplantation might improve prognostic accuracy and outcomes.[27]

Another critical dimension of the pre-transplant assessment in ACLF patients is oncologic screening, which was markedly reduced compared with other clinical settings. Although certain investigations such as thoraco-abdominal CT scans are performed as part of routine ICU imaging, specific cancer screening procedures—particularly for age-related malignancies—were significantly underused in patients with ACLF with significant differences compared with both OutPat and Hosp patients. Such practices likely reflect both logistical constraints and prioritization of time-sensitive interventions in ICU settings. Although this raises concerns about the potential for undiagnosed malignancies that could represent absolute contraindications to LT, current data do not suggest an increased long-term cancer risk in this population.[6,19] We did not observe an increased incidence of *de novo* malignancies in patients who were placed on the WL after ACLF onset. This observation supports a pragmatic approach that prioritizes timely listing, especially in critically ill patients with ACLF. We hence would recall the need for post-transplant vigilance and possibly delayed cancer screening strategies once the patient has stabilized. Future guidelines could also explore whether targeted, risk-adapted oncological screening—rather than complete omission—could offer a feasible compromise in this high-risk population.

Overall, nutritional assessment appears to be underutilized in patients with ACLF. Two main considerations may explain this observation. First, the urgency to offer LT as a life-saving intervention in an otherwise terminal clinical trajectory may lead to the deprioritization of other key parameters that are more systematically evaluated in elective transplant candidates. Second, and perhaps more importantly, the very short median interval between listing and transplantation, together with the current lack of evidence that a brief course of enteral nutritional support improves post-transplant outcomes, may discourage clinicians from performing comprehensive nutritional assessments or initiating targeted nutritional interventions. Future studies are therefore needed to identify relevant surrogate metabolic or nutritional markers and to determine whether optimized perioperative nutritional strategies could contribute to improved outcomes in this high-risk population.

The main limitations of this survey are inherent to its design and to the selection of participants, who were all centers with recognized expertise in the management of patients with ACLF. In addition, the semi-structured nature of the survey inevitably introduces reporting bias and may not fully reflect real-world practice. Although respondents were asked to report institutional practices, individual perceptions and recall bias may have influenced some responses. Moreover, the survey focused exclusively on evaluations performed before registration on the WL and did not capture assessments conducted after registration, post-transplant management, or clinical outcomes in relation to specific pre-LT strategies. Finally, most participating centers were French, which may limit the generalizability of the findings. To address this issue, we performed comparative analyses between French and non-French LT centers. This survey reveals significant differences in practices between centers. Cardiovascular screening differs between French and other European LT centers. Addiction assessment differs both between French and other European LT centers, and French LT and non-LT centers. However, the number of non-French centers were limited. Therefore, further dedicated studies will be necessary to assess the specific characteristics of pre-LT workup in patients with ACLF in each country, taking into account the allocating rules of liver grafts, and the duration on the WL. Such studies will facilitate to harmonize pre-LT evaluation in dedicated clinical practice guidelines strategies to ensure equitable access to LT in this high-risk population.

In conclusion, in patients with ACLF-ICU evaluated for LT, pre-transplantation workup is abbreviated with critical gaps—particularly in cardiological and addiction and psychological assessments. Our study suggests avenues for improving risk stratification and harmonizing practices. Future prospective studies are warranted to evaluate the impact of targeted pre-LT assessments in patients with ACLF on post-transplant outcomes, including cardiovascular events and alcohol relapse.

Affiliations

[1]*Service d'Hépato-Gastroentérologie, FHU Suport, Hôpital Trousseau, CHRU de Tours, Chambray-lès-Tours, France;* [2]*Liver Department, Rennes University Hospital and Rennes University, Rennes, France;* [3]*Service d'Hépato-Gastroentérologie, Clermont Ferrand University Hospital, Clermont Ferrand, France;* [4]*Department of*

Hepatology and Liver Transplantation, Queen Elizabeth University Hospital, Birmingham, UK; [5]Service d'Hépato-Gastroentérologie, Hôpital La Croix Rousse, Hospices civils de Lyon, Lyon, France; [6]Service d'Hépato-Gastroentérologie, Hôpital Pasteur, CHU de Nice, Nice, France; [7]Service d'Hépato-Gastroentérologie, Hôtel Dieu Hospital, Nantes University Hospital, Nantes, France; [8]Service d'Hépato-Gastroentérologie, Centre Hospitalier Sud Francilien, Corbeil Essonnes, France; [9]Service d'Hépatologie, Nancy University Hospital, Nancy, France; [10]Liver Department, King's College Hospital, London, UK; [11]Service d'Hépato-Gastroentérologie, Hôpital Rangueil, Toulouse University Hospital, Toulouse, France; [12]Service d'Hépatologie, Hôpital Saint-Antoine, APHP and Sorbonne University, Paris, France; [13]Service d'Hépato-Gastroentérologie, Groupe Hospitalier Sud de l'Oise, Creil, France; [14]Liver Department, Curry Cabral Hospital, Lisbon, Portugal; [15]Service d'Hépato-Gastroentérologie, Hôpital Dupuytren, Limoges University Hospital, Limoges, France; [16]Centre Hépato-Biliaire, Hôpital Paul Brousse, Inserm U1193, Université Paris-Saclay, Paris, France; [17]Hepatology and Liver Transplantation Unit, Hospital Universitario y Politécnico La Fe, IIS La Fe, Valencia. Ciberehd, Instituto de Salud Carlos III, Madrid, Spain; [18]Service d'Hépato-Gastroentérologie, Hôptal Pitié Salpétrière, APHP and Sorbonne University, Paris, France; [19]Service d'Hépato-Gastroentérologie, Hôpital Michallon, Grenoble Alpes University Hospital, Grenoble, France; [20]Liver Transplant Unit, Department of Hepatology, Hospital Clinic, University of Barcelona, IDIBAPS, CIBERehd, Barcelona, Spain; [21]Liver Department, Erasmus MC University Medical Center, Rotterdam, The Netherlands; [22]Service d'Hépato-Gastroentérologie, Lille University Hospital, Lille, France; [23]Service d'Hépato-Gastroentérologie, Hôpital Henri Mondor, APHP, Paris-Est Créteil University, Paris, France; [24]Liver Department, Charité - Universitaetsmedizin Berlin, Berlin, Germany; [25]Service d'Hépato-Gastroentérologie, Strasbourg University Hospital, Strasbourg, France; [26]Service d'Hépato-Gastroentérologie, Hôpital Beaujon APHP and Université Paris-Cité, Clichy, France; [27]Service d'Hépato-Gastroentérologie, Centre Hospitalier Intercommunal Villeneuve-Saint-Georges, Villeneuve-Saint-Georges, France; [28]Unité d'Hépatologie, Hôpital de la Timone, AP-HM, Marseille, France; [29]Service d'Hépato-Gastroentérologie, Hôpital Christian Cabrol, CHRU de Reims, Reims, France; [30]Service d'Hépato-Gastroentérologie, Hôpital Charles-Nicolle CHU de Rouen, Rouen, France; [31]Transplantation Department and Gastroenterology, Hepato-Pancreatology and Digestive Oncology Department, H.U.B. Hôptal Erasme, Université Libre de Bruxelles, Brussels, Belgium; [32]Liver Department, Royal Free Hospital, London, UK; [33]Service d'Hépato-Gastroentérologie, Centre Hospitalier Départemental Vendée, La Roche sur Yon, France; [34]Service d'Hépato-Gastroentérologie, CHU d'Angers, Angers, France; [35]Service d'Hépato-Gastroentérologie, CHU François Mitterrand, Dijon, France; [36]Service d'Hépato-Gastroentérologie, CHU de Bordeaux, Bordeaux, France; [37]Division of Transplantation, Department of Surgery, Geneva University Hospitals, Geneva, Switzerland; [38]Hospital Universitario Ramón y Cajal, IRYCIS, CIBEREHD, Alcalá University, Madrid, Spain; [39]Service d'Hépato-Gastroentérologie, Centre Hospitalier du Mans, Le Mans, France; [40]Dipartimento di Medicina Translazionale e di Precisione, Policlinico Universitario Umberto1, Sapienza Universita' di Roma, Rome, Italy; [41]Service d'Hépato-Gastroentérologie, CHU de Montpellier, Montpellier, France; [42]Service d'Hépato-Gastroentérologie, Hôpital Côte de Nacre, CHU de Caen, Caen, France; [43]S.C. Epatologia e Gastroenterologia, Dipartimento Medico Polispecialistico, ASST Grande Ospedale Metropolitano Niguarda, Milan, Italy; [44]Medizinische Klinik B, Universitätsklinikum Münster, Münster, Germany; [45]Service d'Hépato-Gastroentérologie, Hôpital Cavale Blanche Hospital, CHU de Brest, Brest, France; [46]Division of Gastroenterology and Hepatology – Dept of Medicine III, MedUni Vienna, Vienna, Italy; [47]Service d'Hépato-Gastroentérologie, CHU de Poitiers, Poitiers, France; [48]Service d'Hépato-Gastroentérologie, Centre Hospitalier Intercommunal de Créteil, Créteil, France; [49]Service d'Hépato-Gastroentérologie, CHU d'Amiens-Picardie, Amiens, France; [50]Service d'Hépato-Gastroentérologie, Centre Hospitalier de Valenciennes, Valenciennes, France; [51]Service d'Hépato-Gastroentérologie, Hôpital Jean Minjoz CHU de Besançon, Besançon, France; [52]Liver Department, Padua University Hospital, Padua, Italy

Abbreviations

ACLF, acute-on-chronic liver failure; ACLF-ICU, acute-on-chronic liver failure admitted to intensive care unit; AUDIT, Alcohol Use Disorders Identification Test; CACS, coronary artery calcium scan; CAD-LT, coronary artery disease-liver transplantation score; CCTA, coronary computed tomography angiography; CLIF, chronic liver failure-C; CT, computed tomography, cardiothoracic scan; DSM, *Diagnostic and Statistical Manual of Mental Disorders*; ECG, electrocardiogram; ENT, ear, nose, and throat; GI, gastrointestinal; Hosp, hospitalized patients in conventional ward without ACLF; ICU, intensive care unit; LT, liver transplant, liver transplantation; MACE, major adverse cardiovascular events; MELD, model for end-stage liver disease; ORL, oto-rhino-laryngology; OutPat, outpatients; PSA, prostate-specific antigen; TTE, transthoracic echocardiography; WL, waiting list; NS, non-significant.

Financial support

No financial support was received to produce this manuscript.

Conflicts of interest

The authors declare no conflicts of interest.

Please refer to the accompanying ICMJE disclosure forms for further details.

Authors' contributions

Conceptualization (lead): FA, LE. Conceptualization (supporting): CB. Formal analysis (lead): FA, LE, CB. Data acquisition (lead): CB. Data acquisition (supporting): all authors.

Writing—original draft (lead): FA, LE, CB. Writing—review and editing (lead): FA, LE.

Writing—review and editing (supporting): all authors.

Data availability

The data that support the findings of this study are available from the corresponding author upon reasonable request.

Supplementary data

Supplementary data to this article can be found online at https://doi.org/10.1016/j.jhepr.2026.101794.

References

Author names in bold designate shared co-first authorship.

[1] **Goldberg D**, Mantero A, Newcomb C, et al. Development and validation of a model to predict long-term survival after liver transplantation. Liver Transpl 2021;27:797–807.
[2] **Rana A**, Ackah RL, Webb GJ, et al. No gains in long-term survival after liver transplantation over the past three decades. Ann Surg 2019;269:20–27.
[3] Agence de la Biomedecine. Rapport médical et scientifique. Greffe hépatique; 2021.
[4] **Arroyo V**, Moreau R, Jalan R. Acute-on-chronic liver failure. N Engl J Med 2020;382:2137–2145.
[5] **Artru F**, **Trovato F**, Morrison M, et al. Liver transplantation for acute-on-chronic liver failure. Lancet Gastroenterol Hepatol 2024;9:564–576.
[6] **Artru F**, Sacleux S-C, Ursic-Bedoya J, et al. Long-term outcome following liver transplantation of patients with ACLF grade 3. J Hepatol 2025;82:62–71.
[7] **Erard-Poinsot D**, Dharancy S, Hilleret M-N, et al. Natural history of recurrent alcohol-related cirrhosis after liver transplantation: fast and furious. Liver Transpl 2020;26:25–33.
[8] European Association for the Study of the Liver. EASL Clinical Practice Guidelines on liver transplantation. J Hepatol 2024;81:1040–1086.
[9] **Sérée O**, Altieri M, Guillaume E, et al. Longterm risk of solid organ de novo malignancies after liver transplantation: a French national study on 11,226 patients. Liver Transpl 2018;24:1425–1436.
[10] **Lai JC**, Shui AM, Duarte-Rojo A, et al. Frailty, mortality, and health care utilization after liver transplantation: from the Multicenter Functional Assessment in Liver Transplantation (FrAILT) study. Hepatology 2022;75:1471–1479.
[11] **Barman PM**, **VanWagner LB**. Cardiac risk assessment in liver transplant candidates: current controversies and future directions. Hepatology 2021;73:2564–2576.
[12] **Artzner T**, **Bernal W**, **Belli LS**, et al. Location and allocation: inequity of access to liver transplantation for patients with severe acute-on-chronic liver failure in Europe. Liver Transpl 2022;28:1429–1440.
[13] **Sacleux S-C-**, Ichaï P, Coilly A, et al. Liver transplant selection criteria and outcomes in critically ill patients with ACLF. JHEP Rep 2024;6:100929.
[14] **Ideo F**, Iegri C, Vigano' M, et al. Liver transplant in ACLF and ALD: an effective option with limited access? Preliminary results of a single center experience. Dig Liver Dis 2024;56:S73.
[15] **Kim D-S**, Yoon Y-I, Kim BK, et al. Asian Pacific Association for the Study of the Liver clinical practice guidelines on liver transplantation. Hepatol Int 2024;18:299–383.

[16] **Martin P**, DiMartini A, Feng S, et al. Evaluation for liver transplantation in adults: 2013 practice guideline by the American Association for the Study of Liver Diseases and the American Society of Transplantation. Hepatology 2014;59:1144–1165.
[17] **John BV**, Schwartz K, Scheinberg AR, et al. Evaluation within 30 days of referral for liver transplantation is associated with reduced mortality: a multicenter analysis of patients referred within the VA health system. Transplantation 2022;106:72–84.
[18] European Association for the Study of the Liver. EASL Clinical Practice Guidelines on acute-on-chronic liver failure. J Hepatol 2023;79:461–491.
[19] **Sundaram V**, **Mahmud N**, Perricone G, et al. Longterm outcomes of patients undergoing liver transplantation for acute-on-chronic liver failure. Liver Transpl 2020;26:1594–1602.
[20] **Harinstein ME**, Gandolfo C, Gruttadauria S, et al. Cardiovascular disease assessment and management in liver transplantation. Eur Heart J 2024;45:4399–4413.
[21] **Harrington CR**, Levy P, Cabrera E, et al. Evolution of pretransplant cardiac risk factor burden and major adverse cardiovascular events in liver transplant recipients over time. Liver Transpl 2023;29:581–590.
[22] **Pagano G**, Sastre L, Blasi A, et al. CACS, CCTA and mCAD-LT score in the pre-transplant assessment of coronary artery disease and the prediction of post-transplant cardiovascular events. Liver Int 2024;44:1912–1923.
[23] **Rachwan RJ**, **Kutkut I**, **Timsina LR**, et al. CAD-LT score effectively predicts risk of significant coronary artery disease in liver transplant candidates. J Hepatol 2021;75:142–149.
[24] **Tang H**, **Shi C**, Chapin S, et al. Predictive performance of CAR-OLT and CAD-LT scores for post-transplant adverse cardiovascular events in a large transplant center. Liver Transpl 2023;29:1008–1011.
[25] **Mathurin P**, Moreno C, Samuel D, et al. Early liver transplantation for severe alcoholic hepatitis. N Engl J Med 2011;365:1790–1800.
[26] **Louvet A**, Labreuche J, Moreno C, et al. Early liver transplantation for severe alcohol-related hepatitis not responding to medical treatment: a prospective controlled study. Lancet Gastroenterol Hepatol 2022;7:416–425.
[27] **Daniel J**, Dumortier J, Del Bello A, et al. Integrating an addiction team into the management of patients transplanted for alcohol-associated liver disease reduces the risk of severe relapse. JHEP Rep 2023;5:100832.

Keywords: Cirrhosis; Liver transplantation; Acute-on-chronic liver failure; Alcohol-related hepatitis; Pre-transplant workup; Organ failure.

Received 19 June 2025; received in revised form 9 February 2026; accepted 16 February 2026; Available online 26 February 2026

Journal of Hepatology, Volume *8*

Supplemental information

Pre-liver transplant assessment of patients with acute-on-chronic liver failure: An international survey

Claire Bellec, Anea Augé, Sébastien L'Hermite, Romain Moirand, Armand Abergel, Matthew Amstrong, Teresa Antonini, Rodolphe Anty, Marion Khaldi, Aurore Baron, Mouni Bensenane-Oussalah, William Bernal, Christophe Bureau, Nicolas Carbonell, Jean-François Cadranel, Filipe Cardoso, Paul Carrier, Audrey Coilly, Isabel Conde, Filomena Conti, Agnes Bonadona, Gonzalo Crespo, Sarwa Darwish Murad, Sébastien Dharancy, Christophe Duvoux, Cornelius Engelmann, François Faitot, Claire Francoz, Armand Garioud, René Gerolami, Elia Gigante, Odile Goria, Thierry Gustot, Brian Hogan, Ludovic Lagin, Adrien Lannes, Marianne Latournerie, Victor de Ledinghen, Giulia Magini, Martin Mateo, Arnaud Maurin, Manuela Merli, Magdalena Meszaros, Georges-Philippe Pageaux, Claire Perignon, Giovanni Perricone, Michael Praktiknjo, Noemi Reboux, Thomas Reiberger, Valentin Rolle, Isabelle Rosa, Ruxandra Sarba, Faustine Wartel, Delphine Verhoeven Weil, Alberto Zanetto, Laure Elkrief, and Florent Artru

Pre-liver transplant assessment of patients with acute-on-chronic liver failure: An international survey

Claire Bellec, Anéa Augé, Sébastien L'Hermite, Romain Moirand, Armand Abergel, Matthew Amstrong, Teresa Antonini, Rodolphe Anty, Marion Khaldi, Aurore Baron, Mouni Bensenane-Oussalah, William Bernal, Christophe Bureau, Nicolas Carbonell, Jean-François Cadranel, Filipe Cardoso, Paul Carrier, Audrey Coilly, Isabel Conde, Filomena Conti, Agnes Bonadona, Gonzalo Crespo, Sarwa Darwish Murad, Sébastien Dharancy, Christophe Duvoux, Cornelius Engelmann, François Faitot, Claire Francoz, Armand Garioud, René Gerolami, Elia Gigante, Odile Goria, Thierry Gustot, Brian Hogan, Ludovic Lagin, Adrien Lannes, Marianne Latournerie, Victor de Ledinghen, Giulia Magini, Martin Mateo, Arnaud Maurin, Manuela Merli, Magdalena Meszaros, Georges-Philippe Pageaux, Claire Perignon, Giovanni Perricone, Michael Praktiknjo, Noemi Reboux, Thomas Reiberger, Valentin Rolle, Isabelle Rosa , Ruxandra Sarba, Faustine Wartel, Delphine Verhoeven Weil, Alberto Zanetto, Laure Elkrief, Florent Artru

Table of contents

Table S1……………………………………………………………………………………….2

Table S2……………………………………………………………………………………….4

Table S3……………………………………………………………………………………...10

Table S4……………………………………………………………………………………...16

Table S1. List of the participating centers.

	Country	Transplant / non transplant center	Academic / Non academic hospital
Clermont-Ferrand University Hospital	France	Transplant	Academic
Hospices civils de Lyon, Lyon University Hospital	France	Transplant	Academic
Pasteur Hospital of Nice, Nice University Hospital	France	Transplant	Academic
Pierre-Paul Riquet Hospital of Toulouse, Toulouse University Hospital	France	Transplant	Academic
Paul Brousse Hospital, Paris-Saclay University	France	Transplant	Academic
Pitié Salpétrière Charles Foix University Hospital	France	Transplant	Academic
Michallon Hospital, Grenoble Alpes University Hospital	France	Transplant	Academic
Bordeaux University Hospital	France	Transplant	Academic
Lille University Hospital	France	Transplant	Academic
Strasbourg University Hospital	France	Transplant	Academic
Beaujon Hospital, Paris Cité University	France	Transplant	Academic
Marseille Timone University Hospital	France	Transplant	Academic
Montpellier University Hospital	France	Transplant	Academic
Jean Minjoz Hospital, Besançon University Hospital	France	Transplant	Academic
Trousseau Hospital, Tours University Hospital	France	Transplant	Academic
Pontcahillou Hospital, Rennes University Hospital	France	Transplant	Academic
Hôtel Dieu Hospital, Nantes University Hospital	France	Non transplant	Academic
Saint Antoine Hospital, Sorbonne University	France	Non transplant	Academic
Dupuytren Hospital, Limoges University Hospital	France	Non transplant	Academic
Christian Cabrol Hospital, Reims University Hospital	France	Non transplant	Academic
Angers University Hospital	France	Non transplant	Academic
François-Mitterand Hospital, Dijon University Hospital	France	Non transplant	Academic
Avicenne Hospital, Bobigny University	France	Non transplant	Academic
Côte de Nacre Hospital, Caen Normandie University Hospital	France	Non transplant	Academic
Cavale Blanche Hospital, Brest University Hospital	France	Non transplant	Academic
Poitiers University Hospital	France	Non transplant	Academic
Amiens-Picardie University Hospital	France	Non transplant	Academic
Charles-Nicolle Hospital, Rouen University Hospital	France	Non transplant	Academic

Henri-Mondor Hospital, Paris-Est Créteil University	France	Non transplant	Academic
Brabois Hospital, Nancy University Hospital	France	Non transplant	Academic
Créteil Intercommucal Hospital	France	Non transplant	Non academic
Sud Francilien Hospital, Corbeil-Essonnes	France	Non transplant	Non academic
Sud de l'Oise Public Hospital Group, Creil	France	Non transplant	Non academic
Villeuneuve-Saint-Georges Intercommunal Hospital	France	Non transplant	Non academic
Valenciennes Hospital	France	Non transplant	Non academic
Le Mans Hospital	France	Non transplant	Non academic
Départemental Vendée Hospital , La Roche sur Yon	France	Non transplant	Non academic
University Hospital Birmingham	United Kingdom	Transplant	Academic
Medical University of Vienna	Austria	Transplant	Academic
King's College Hospital, London	United Kingdom	Transplant	Academic
Curry Cabral Hospital, Lisbon	Portugal	Transplant	Academic
Hospital Universitario y Politecnico La Fe, Valence	Spain	Transplant	Academic
Hospital Clínic de Barcelona	Spain	Transplant	Academic
Charité - Universitaetsmedizin Berlin	Germany	Transplant	Academic
Erasme Hospital, Bruxelles University Hospital	Belgium	Transplant	Academic
Royal Free Hospital, London	United Kingdom	Transplant	Academic
Geneve University Hospital	Switzerland	Transplant	Academic
Ramón y Cajal University Hospital, Madrid	Spain	Transplant	Academic
Rome University Hospital	Italy	Transplant	Academic
Erasmus MC University Medical , Rotterdam	Netherlands	Transplant	Academic
ASST Grande Ospedale Metropolitano Niguarda, Milan	Italy	Transplant	Academic
Münster University Hospital	Germany	Transplant	Academic
Padua University Hospital	Italy	Transplant	Academic

Table S2: Answers to the questionnaire from the 53 centers according to patient's setting at the time of pre-transplant evaluation.

Question	Outpatients (OutPat)	Patients hospitalized in liver ward without ACLF (Hosp)	Patients with ACLF in ICU (ACLF-ICU)	p value ACLF *vs.* OutPat	p value ACLF *vs.* Hosp
**Cardiac and respiratory assessment**					
**Do you systematically perform an ECG?**					
Yes	49 (92.5)	52 (98.1)	52 (98.1)	0.17	1
No	4 (7.5)	1 (1.9)	1 (1.9)		
**Do you systematically perform a TTE?**					
Yes	53 (100)	52 (98.1)	53 (100)	1	0.32
No	0 (0)	1 (1.9)	0 (0)		
**Do you systematically request a stress exam?**					
Yes	11 (20.8)	9 (17.0)	1 (1.9)	0.002	0.008
No	42 (79.2)	44 (83.0)	52 (98.1)		
**Do you perform a stress exam in case of cardiovascular risk factors?**					
Yes	22 (41.5)	24 (45.3)	5 (9.4)	0.0002	<0.0001
No	31 (58.5)	29 (54.7)	48 (90.6)		
**Do you systematically perform a CCTA or a CACS?**					
Yes	4 (7.5)	3 (5.7)	0 (0)	0.04	0.08
No	49 (92.5)	50 (94.3)	53 (100)		
**Do you perform a CCTA or a CACS in case of cardiovascular risk factors?**					
Yes	11 (20.8)	11 (20.8)	2 (3.8)	0.008	0.008
No	42 (79.2)	42 (79.2)	51 (96.2)		
**When do you perform a coronarography?**					
- In case of cardiovascular risk factors	18 (34.0)	16 (30.2)	12 (22.6)	0.19	0.38
- In case of symptoms	27 (50.9)	23 (43.4)	31 (58.5)	0.43	0.12
- In case of abnormal noninvasive tests	40 (75.5)	35 (66.0)	22 (41.5)	0.0004	0.01
**What percentage of patients undergo a right-heart catheterism?**					

- < 25% patients<br>- > 25% of patients	49 (92.5)<br>4 (7.5)	47 (88.7)<br>6 (11.3)	51 (96.2)<br>2 (3.8)	0.41	0.17
**When do you perform a right-heart catheterism in your center?**					
- Always	2 (3.8)	2 (3.8)	1 (1.9)	0.56	0.56
- In case of abnormal TTE results	51 (96.2)	50 (94.3)	51 (96.2)	1	0.65
- In case of symptoms	17 (32.1)	16 (30.2)	10 (18.9)	0.12	0.18
**When do you perform a doppler of supra-aortic trunks?**					
- Always<br>- In case of risk factors<br>- Never	20 (37.8)<br>16 (30.2)<br>8 (15.1)	23 (43.4)<br>26 (49.1)<br>19 (35.8)	10 (18.9)<br>11 (20.8)<br>26 (49.1)	0.008	<0.0001
**When do you request a cardiologist consultation?**					
- Always<br>- In case of risk factors<br>- Never	27 (50.9)<br>23 (43.4)<br>3 (5.7)	24 (45.3)<br>24 (45.3)<br>5 (9.4)	17 (32.1)<br>19 (35.8)<br>17 (32.1)	0.002	<0.0001
**What respiratory exam(s) do you systematically request?**					
- Thoracic CT scan	49 (92.5)	50 (94.3)	53 (100)	0.04	0.08
- Respiratory function test	47 (88.7)	40 (75.5)	13 (24.5)	<0.0001	<0.0001
- 6 minutes' walk test	7 (13.2)	4 (7.5)	0 (0)	0.006	0.04
- pneumologist consultation	10 (18.9)	8 (15.1)	3 (5.7)	0.04	0.11
**Addictology assessment**					
**How many patients are assessed by a senior addictologist?**					
- < 25% patients<br>- 26-50% patients<br>- 51-75% patients<br>- > 75% patients	3 (5.7)<br>9 (17.0)<br>8 (15.1)<br>33 (62.3)	5 (13.5)<br>2 (5.4)<br>4 (10.8)<br>26 (70.3)	8 (21.6)<br>4 (10.8)<br>6 (16.2)<br>19 (51.4)	0.01	0.39
**If the first addictology assessment was realized in a nontransplant center, is it systematically performed again in the transplantation center?**					
- Yes	38 (71.7)	38 (71.7)	36 (67.9)	0.67	0.67

- No	15 (28.3)	15 (28.3)	17 (32.1)		
**Apart from alcohol-associated hepatitis, what is the minimal required duration of alcohol cessation to consider liver transplantation?**					
- ≥ 6 months	16 (30.1)	8 (15.2)	5 (9.4)		
- 3-6 months	19 (35.9)	20 (37.7)	12 (22.6)		
- 1-3 months	1 (1.9)	4 (7.5)	2 (3.8)	0.008	<0.0001
- < 1 month	0	0	1 (1.9)		
- Case by case decision	17 (32.1)	21 (39.6)	33 (62.3)		
**Decisive components to predict alcohol relapse risk after transplantation? (1- most important, 8- less important)**					
- Addiction diagnosis according to DSM V	4 (1-8)	5 (2-8)	5 (2-8)	0.73	0.92
- Former failure of withdrawal cure	3 (2-6)	3 (2-6)	3 (2-5)	0.29	0.43
- Polyaddiction	4 (2-6)	4 (2-7)	4 (3-6)	0.45	0.45
- Psychiatric comorbidities	4 (3-7)	4 (2-6)	4 (3-6)	0.45	0.88
- Socio-professional insertion	5 (4-6)	5 (4-5)	4 (3-5)	0.36	0.75
- Relatives' quality	5 (4-6)	5 (4-6)	5 (3-6)	0.96	0.79
- Insight of their alcohol use disorder	3 (2-6)	4 (2-7)	5 (3-7)	0.17	0.63
- Addictologist follow-up	6 (3-7)	6 (3-7)	6 (2-7)	0.90	0.98
- **Which score(s) do you routinely use for your addictology assessment?**					
- AUDIT score	47 (88.7)	45 (84.9)	47 (88.7)	1.0	0.57
- HRAR score	7 (13.2)	9 (17.0)	8 (15.1)	0.78	0.79
- SALT score	5 (9.4)	7 (13.2)	6 (11.3)	0.75	0.77
**In which circumstances do you support your addictology assessment by questioning the relatives?**					
- Always	32 (60.4)	37 (69.8)	38 (71.7)	0.22	0.81
- In case of patient's encephalopathy/intubation	17 (32.1)	13 (24.5)	18 (34.0)	0.84	0.29
**Who is questioned in the family when this is done?**					
- The partner	53 (100)	53 (100)	52 (98.1)	0.31	0.31
- Children and parents	45 (84.9)	49 (92.5)	50 (94.3)	0.11	0.70

- Friends /colleagues<br>- The general practitioner	4 (7.5)<br>34 (64.1)	5 (9.4)<br>32 (60.4)	8 (15.1)<br>37 (69.8)	0.22<br>0.54	0.37<br>0.31
**How often do you perform blood, urinary or capillary screening tests?**<br>- < 25% patients<br>- 26 -50% patients<br>- 51 -75% patients<br>- > 75% patients	18 (34.0)<br>10 (18.8)<br>7 (13.2)<br>18 (34.0)	23 (43.4)<br>5 (9.4)<br>8 (15.1)<br>17 (32.1)	31 (58.5)<br>6 (11.3)<br>2 (3.8)<br>14 (26.4)	0.052	0.16
**Which biological or urinary test is most often performed in your center?**<br>- Carboxydate Deficience Transferrine<br>- Phosphatydyléthanol<br>- Urinary ethyl glucuronide<br>- Capillary ethyl glucoronide	15 (28.3)<br>20 (37.7)<br>14 (26.4)<br>4 (7.5)	14 (26.4)<br>19 (35.9)<br>17 (32.1)<br>3 (5.7)	17 (32.1)<br>17(32.1)<br>16 (30.2)<br>3 (5.7)	0.89	0.77
**Do you always take into account the addictologist's opinion?**<br>- Yes<br>- No	43 (81.1)<br>10 (18.9)	43 (81.1)<br>10 (18.9)	42 (79.3)<br>11 (20.7)	0.81	0.81
**Oncologic assessment<br>In patients above 50 years old, what exams do you perform?**					
**When do you perform a colonoscopy**<br>- In all patients<br>- In case of risk factors of colorectal neoplasm	34 (64.1)<br>17 (32.1)	26 (49.1)<br>16 (30.2)	9 (16.7)<br>13 (24.5)	<0.0001<br>0.39	0.0004<br>0.51
**Do you systematically perform an upper GI endoscopy?**<br>- Yes<br>- No	49 (92.5)<br>4 (7.5)	43 (81.1)<br>10 (18.9)	34 (64.1)<br>19 (35.9)	0.0004	0.05
**Do you systematically perform a CT scan of the abdomen and pelvis?**<br>- Yes<br>- No	49 (92.5)<br>4 (7.5)	49 (92.5)<br>4 (7.5)	53 (100)<br>0 (0)	0.04	0.04
**Do you systematically perform a PSA dosage in men?**<br>- Yes	42 (79.3)	41 (77.4)	36 (67.9)	0.19	0.28

- No	11(20.7)	12 (22.6)	17 (32.1)		
**Do you systematically perform a mammography, pap smear, and gynecologic exam for women?**<br>- Yes<br>- No	 <br><br>43 (81.1)<br>10 (18.9)	 <br><br>35 (66.0)<br>18 (34.0)	 <br><br>16 (30.2)<br>37 (69.8)	 <br><br><0.0001	 <br><br>0.0002
**Do you systematically perform an ENT consultation?**<br>- Yes<br>- No	 <br>39 (73.6)<br>14 (26.4)	 <br>34 (64.1)<br>19 (35.9)	 <br>19 (35.9)<br>34 (64.1)	 <br>0.0001	 <br>0.004
**Do you systematically perform a dermatology consultation?**<br>- Yes<br>- No	 <br>25 (47.2)<br>23 (52.8)	 <br>23 (43.4)<br>30 (56.6)	 <br>9 (17.0)<br>44 (83.0)	 <br>0.0009	 <br>0.004
**Nutritional assessment**					
**When do you request nutritionist or dietitian consultation?**<br>- Systematically<br>- In case of clinical malnutrition<br>- Never	 <br>16 (29.7)<br>37 (70.3)<br>0 (0)	 <br>26 (49.1)<br>15 (28.3)<br>12 (22.6)	 <br>19 (35.8)<br>15 (28.3)<br>19 (35.8)	 <br><br><0.0001	 <br><br>0.003
**When do you perform a screening for malnutrition using questionnaires such as SGA, RFH-NPT**<br>- Systematically<br>- In case of clinical malnutrition	 <br><br>10 (18.9)<br>16 (30.2)	 <br><br>13 (24.5)<br>11 (20.8)	 <br><br>9 (17.0)<br>8 (15.1)	 <br><br>0.80<br>0.06	 <br><br>0.34<br>0.45
**When do you perform a screening for sarcopenia using psoas measurement by CT scan?**<br>- Systematically<br>- In case of clinical malnutrition	 <br><br>4 (7.6)<br>7 (13.2)	 <br><br>4 (7.6)<br>6 (11.3)	 <br><br>5 (9.4)<br>8 (15.1)	 <br><br>1<br>0.78	 <br><br>0.73<br>0.57
**When do you perform a screening for frailty using dedicated tests (for example LFI)**?<br>- Systematically<br>- In case of clinical malnutrition	 <br>14 (26.4)<br>23 (43.4)	 <br>11 (20.8)<br>24 (45.3)	 <br>8 (15.1)<br>6 (11.3)	 <br>0.16<br>0.0002	 <br>0.45<br>0.0001
**General considerations**					

**In your experience, what is the timeframe in days between initiation of LT assessment and listing?**	45 (30-90)	18 (14-30)	7 (3-10)	<0.0001	<0.0001
**What proportion of ACLF patients develop ACLF while already listed for LT, with a complete pre-transplant workup?**<br>- < 25% patients<br>- 26 -50% patients<br>- 51 -75% patients<br>- 75% patients			 38 (71.7)<br>11 (20.8)<br>3 (5.7)<br>1 (1.9)	N/A	N/A
**How do you describe the pre-transplant workup in ACLF patients in ICU, as compared with other situations?**<br>- Similar to other situations<br>- Abbreviated workup to allow for a quick listing assuming that it will still be able to identify definitive contraindications to LT<br>- Abbreviated workup to allow for a quick listing with the risk of not identifying definitive contraindications to LT<br>- More comprehensive workup to secure LT results in this very sick population			 1 (1.9)<br>39 (73.6)<br>12 (22.6)<br>1 (1.9)	N/A	N/A

Abbreviations : ACLF: Acute On Chronic Liver Failure, AUD: alcohol use disorder, AUDIT: Alcohol Use Disorders Identification Test, CCAS: coronary artery calcium scan, CCTA: coronary CT angiography, DSM: Diagnostic and Statistical Manual of Mental Disorders, ECG: electrocardiogram, EGD: oesophagograstroduodenoscopy, ENT: Ear-Nose-Throat, HRAR: High-Risk Alcoholism Relapse, Hosp: hospitalized patients without ALCF; LFI: liver frailty index, LT: liver transplantation, OutPat: outpatients, PSA: Prostatic Specific Antigen, RFH-NPT: Royal Free Hospital nutritional prioriziting tool, SALT: Sustained Alcohol Use After Early Liver Transplant, SGA: subjective global assessment, TAP: thoraco-abdomino-pelvic, TTE: trans-thoracic echocardiography

Table S3. Answers to the questionnaire regarding pre-transplant evaluation in ACLF-ICU patients among respondents from 32 liver transplant centers according to the country (French *vs.* other countries).

Question	French transplant centers	European transplant centers	p value
**Cardiac and respiratory assessment**			
**Do you systematically perform an ECG?**			
Yes	16 (100)	16 (100)	1
No	0 (0)	0 (0)	
**Do you systematically perform a TTE?**			
Yes	16 (100)	16 (100)	1
No	0 (0)	0 (0)	
**Do you systematically request a stress exam?**			
Yes			
No	0 (0)	0 (0)	1
	16 (100)	16 (100)	
**Do you perform a stress exam in case of cardiovascular risk factors?**			
Yes	0 (0)	3 (25.0)	0.07
No	16 (100)	13 (75.0)	
**Do you systematically perform a CCTA or a CACS?**			
Yes			
No	0 (0)	0 (0)	1
	16 (100)	16 (100)	
**Do you perform a CCTA or a CACS in case of cardiovascular risk factors?**			
Yes	0 (0)	2 (12.5)	0.14
No	16 (100)	14 (87.5)	
**When do you perform a coronary angiography?**			
- In case of cardiovascular risk factors	4 (25.0)	4 (25.0)	1
- In case of symptoms	10 (62.5)	4 (25.0)	0.03
- In case of abnormal noninvasive tests	4 (25.0)	11 (68.8)	0.01
**What percentage of patients undergo a right-heart catheterism?**			

- < 25% patients<br>- > 25% of patients	15 (93.8)<br>1 (6.2)	16 (100)<br>0 (0)	0.31
**When do you perform a right-heart catheterism in your center?**<br>- Always<br>- In case of abnormal TTE results<br>- In case of symptoms	 <br>0 (0)<br>15 (93.8)<br>2 (12.5)	 <br>0 (0)<br>16 (100)<br>2 (12.5)	 <br>1<br>0.31<br>1
**When do you perform a doppler of supra-aortic trunks?**<br>- Always<br>- In case of risk factors<br>- Never	 <br>2 (12.5)<br>4 (25.0)<br>10 (62.5)	 <br>1 (6.3)<br>7 (43.8)<br>8 (50.0)	0.50
**When do you request a cardiologist consultation?**<br>- Always<br>- In case of risk factors<br>- Never	 6 (37.5)<br>3 (18.8)<br>7 (43.7)	 5 (31.3)<br>10 (62.5)<br>1 (6.2)	0.02
**What respiratory exam(s) do you systematically request?**<br>- Thoracic CT scan<br>- Respiratory function test<br>- 6 minutes' walk test<br>- pneumologist consultation	 <br>16 (100)<br>2 (12.5)<br>0 (0)<br>1 (6.3)	 <br>16 (100)<br>3 (18.8)<br>0 (0)<br>1 (6.3)	 <br>1<br>0.62<br>1<br>1
**Addictology assessment**			
**How many patients are assessed by a senior addictologist?**<br>- < 25% patients<br>- 26-50% patients<br>- 51-75% patients<br>- > 75% patients	 <br>2 (12.5)<br>2 (12.5)<br>5 (31.3)<br>7 (43.7)	 <br>6 (37.5)<br>1 (6.3)<br>1 (6.3)<br>8 (50.0)	0.17
**If the first addictology assessment was realized in a nontransplant center, is it systematically performed again in the transplantation center?**<br>- Yes<br>- No	 <br><br>8 (50.0)<br>8 (50.0)	 <br><br>15 (93.8)<br>1 (6.2))	0.006

**Apart from alcohol-related hepatitis, what is the minimal required duration of alcohol cessation to consider liver transplantation?**			
- ≥ 6 months	1 (6.3)	3 (18.8)	
- 3-6 months	0 (0)	4 (25.0)	0.06
- 1-3 months	2 (12.5)	0 (0)	
- < 1 month	0 (0)	1 (6.3)	
- Case by case decision	13 (81.2)	8 (50.0)	
**Decisive components to predict alcohol relapse risk after transplantation? (1- most important, 8- less important)**			
- Addiction diagnosis according to DSM V	3 (1-8)	4 (3-8)	0.23
- Former failure of withdrawal cure	6 (5-8	3 (1-4)	0.0003
- Polyaddiction	6 (3-7)	4 (2-6)	0.07
- Psychiatric comorbidities	4 (3-7)	5 (3-6)	0.87
- Socio-professional insertion	4 (3-5)	6 5-8)	0.003
- Relatives' quality	5 (4-6)	6 (57)	0.12
- Insight of their alcohol use disorder	4 (1-7)	3 (2-6)	0.94
- Addictologist follow-up	5 (2-7)	5 (2-7)	0.78
- **Which score(s) do you routinely use for your addictology assessment?**			
- AUDIT score	14 (87.5)	14 (87.5)	1
- HRAR score	3 (18.8)	2 (12.5)	0.62
- SALT score	1 (6.3)	3 (18.8)	0.29
**In which circumstances do you support your addictology assessment by questioning the relatives?**			
- Always	15 (93.8)	10 (62.5)	0.03
- In case of patient's encephalopathy/intubation	1 (6.2)	8 (50.0)	0.006
**Who is questioned in the family when this is done?**			
- The partner	16 (100)	16 (100)	1
- Children and parents	16 (100)	14 (87.5)	0.14
- Friends /colleagues	4 (25.0)	2 (12.5)	0.37
- The general practitioner	13 (83.8)	6 (37.5)	0.01
**How often do you perform blood, urinary or capillary screening tests?**			
- < 25% patients	9 (56.3)	9 (56.3)	

- 26 -50% patients	3 (18.7)	2 (12.5)	
- 51 -75% patients	0 (0)	1 (6.3)	0.75
- > 75% patients	4 (25.0)	4 (25.0)	
**Which biological or urinary test is most often performed in your center?**			
- Carboxydate Deficience Transferrine	5 (31.2)	5 (31.2)	
- Phosphatydyléthanol	6 (37.5)	1 (6.3)	
- Urinary ethyl glucuronide	4 (25.0)	9 (56.3)	0.14
- Capillary ethyl glucoronide	1 (6.3)	1 (6.3)	
**Do you always take into account the addictologist's opinion?**			
- Yes	12 (75.0)	15 (93.8)	0.14
- No	4 (25.0)	1 (6.2)	
**Oncologic assessment**			
**In patients above 50 years old, what exams do you perform?**			
**When do you perform a colonoscopy**			
- In all patients	1 (6.3)	2 (12.5)	0.54
- in case of risk factors of colorectal neoplasm			
	4 (25.0)	4 (25.0)	1
**Do you systematically perform an upper GI endoscopy?**			
- Yes			
- No	11 (68.8)	7 (43.8)	0.15
	5 (31.2)	9 (56.2)	
**Do you systematically perform a CT scan of the abdomen and pelvis?**			
- Yes	16 (100)	16 (100)	1
- No	0 (0)	0 (0)	
**Do you systematically perform a PSA dosage in men?**			
- Yes	11 (68.8)	9 (56.3)	0.47
- No	5 (31.2)	7 (43.7)	
**Do you systematically perform a mammography, pap smear, and gynecologic exam for women?**			
- Yes	5 (31.2)	4 (25.0)	0.69
- No	11 (68.8)	12 (75.0)	
**Do you systematically perform an ENT consultation?**			

- Yes<br>- No	 6 (37.5)<br>10 (62.5)	 3 (18.8)<br>13 (81.2)	 0.24
**Do you systematically perform a dermatology consultation?**<br>- Yes<br>- No	 <br>3 (18.8)<br>13 (81.2)	 <br>1 (6.3)<br>15 (93.7)	 <br>0.29
**Nutritional assessment**			
**When do you request nutritionist or dietitian consultation?**<br>- Systematically<br>- In case of clinical malnutrition<br>- Never	 <br>7 (43.7)<br>5 (31.3)<br>4 (25.0)	 <br>4 (25.0)<br>4 (25.0)<br>8 (50.0)	 <br><br>0.33
**When do you perform a screening for denutrition using questionnaires such as SGA, RFH-NPT**<br>- Systematically<br>- In case of clinical malnutrition	 <br>2 (12.5)<br>1 (6.3)	 <br>1 (6.3)<br>3 (18.8)	 <br>0.54<br>0.29
**When do you perform a screening for sarcopenia using psoas measurement by CT scan?**<br>- Systematically<br>- In case of clinical malnutrition	 <br>3 (18.8)<br>3 (18.8)	 <br>2(12.5)<br>2 (12.5)	 <br>0.63<br>0.63
**When do you perform a screening for frailty using dedicated tests (for example LFI)**?<br>- Systematically<br>- In case of clinical malnutrition	 <br>3 (18.8)<br>3 (18.8)	 <br>3 (18.8)<br>2 (12.5)	 <br>1<br>0.63
**General considerations**			
**In your experience, what is the timeframe in days between initiation of LT assessment and listing?**	5 (3-7)	5 (3-7)	7 (5-15)
**What proportion of ACLF patients develop ACLF while already listed for LT, with a complete pre-transplant workup?**<br>- < 25% patients<br>- 26 -50% patients<br>- 51 -75% patients<br>- 75% patients	 <br><br>9 (56.3)<br>6 (37.5)<br>1 (6.25)<br>0 (0)	 <br><br>12 (75.0)<br>2 (12.5)<br>1 (6.3)<br>1 (6.3)	 <br><br><br>0.33

**How do you describe the pre-transplant workup in ACLF patients in ICU, as compared with other situations?**			
- Similar to other situations	1 (6.2)	0 (0)	0.52
- Abbreviated workup to allow for a quick listing assuming that it will still be able to identify definitive contraindications to LT	11 (68.8	13 (81.3)	
- Abbreviated workup to allow for a quick listing with the risk of not identifying definitive contraindications to LT	4 (25.0)	3 (18.8)	
- More comprehensive workup to secure LT results in this very sick population	0 (0)	0 (0)	

Abbreviations : ACLF: Acute On Chronic Liver Failure, AUD: alcohol use disorder, AUDIT: Alcohol Use Disorders Identification Test, CCAS: coronary artery calcium scan, CCTA: coronary CT angiography, DSM: Diagnostic and Statistical Manual of Mental Disorders, ECG: electrocardiogram, EGD: oesophagograstroduodenoscopy, ENT: Ear-Nose-Throat, HRAR: High-Risk Alcoholism Relapse, Hosp: hospitalized patients without ALCF; LFI: liver frailty index, LT: liver transplantation, OutPat: outpatients, PSA: Prostatic Specific Antigen, RFH-NPT: Royal Free Hospital nutritional prioriziting tool, SALT: Sustained Alcohol Use After Early Liver Transplant, SGA: subjective global assessment, TAP: thoraco-abdomino-pelvic, TTE: trans-thoracic echocardiography

Table S4: Answers to the questionnaire regarding pre-transplant evaluation in ACLF-ICU patients among respondents from 37 French centers according to the center characteristic (liver transplant vs. non liver transplant center).

Question	French transplant centers	French non transplant centers	p value
**Cardiac and respiratory assessment**			
**Do you systematically perform an ECG?**			
Yes	16 (100)	20 (95.3)	0.38
No	0 (0)	1 (4.7)	
**Do you systematically perform a TTE?**			
Yes	16 (100)	21 (100)	1
No	0 (0)	0 (0)	
**Do you systematically request a stress exam?**			
Yes	0 (0)	1 (4.7)	0.38
No	16 (100)	20 (95.3)	
**Do you perform a stress exam in case of cardiovascular risk factors?**			
Yes	0 (0)	1 (4.7)	0.38
No	16 (100)	20 (95.3)	
**Do you systematically perform a CCTA or a CACS?**			
Yes	0 (0)	0 (0)	1
No	16 (100)	21 (100)	
**Do you perform a CCTA or a CACS in case of cardiovascular risk factors?**			
Yes	0 (0)	0 (0)	1
No	16 (100)	21 (100)	
**When do you perform a coronary angiography?**			
- In case of cardiovascular risk factors	4 (25.0)	4 (19.1)	0.66
- In case of symptoms	10 (62.5)	17 (81.0)	0.21
- In case of abnormal noninvasive tests	4 (25.0)	7 (33.3)	0.58
**What percentage of patients undergo a right-heart catheterism?**			
- < 25% patients	15 (93.8)	20 (95.2)	0.35
- > 25% of patients	1 (6.2)	1 (4.8)	

**When do you perform a right-heart catheterism in your center?**			
- Always	0 (0)	1 (4.8)	0.38
- In case of abnormal TTE results	15 (93.8)	20 (95.2)	0.84
- In case of symptoms	2 (12.5)	6 (28.6)	0.24
**When do you perform a doppler of supra-aortic trunks?**			
- Always	2 (12.5)	5 (23.8)	
- In case of risk factors	4 (25.0)	8 (38.1)	0.33
- Never	10 (62.5)	8 (38.1)	
**When do you request a cardiologist consultation?**			
- Always	6 (37.5)	6 (28.6)	
- In case of risk factors	3 (18.8)	6 (28.6)	0.75
- Never	7 (43.7)	9 (42.8)	
**What respiratory exam(s) do you systematically request?**			
- Thoracic CT scan	16 (100)	21 (100)	1
- Respiratory function test	2 (12.5)	8 (38.1)	0.08
- 6 minutes' walk test	0 (0)	0 (0)	1
- pneumologist consultation	1 (6.3)	1 (4.8)	0.84
**Addictology assessment**			
**How many patients are assessed by a senior addictologist?**			
- < 25% patients	2 (12.5)	6 (28.6)	
- 26-50% patients	2 (12.5)	2 (9.5)	
- 51-75% patients	5 (31.3)	1 (4.8)	0.14
- > 75% patients	7 (43.7)	12 (57.1)	
**If the first addictology assessment was realized in a nontransplant center, is it systematically performed again in the transplantation center?**			
- Yes	8 (50.0)	13 (61.9)	0.47
- No	8 (50.0)	8 (38.1))	
**Apart from alcohol-associated hepatitis, what is the minimal required duration of alcohol cessation to consider liver transplantation?**			

- ≥ 6 months<br>- 3-6 months<br>- 1-3 months<br>- < 1 month<br>- Case by case decision	1 (6.3)<br>0 (0)<br>2 (12.5)<br>0 (0)<br>13 (81.2)	1 (4.8)<br>8 (38.1)<br>0 (0)<br>0 (0)<br>12 (57.1)	0.02
**Decisive components to predict alcohol relapse risk after transplantation? (1- most important, 8- less important)**			
- Addiction diagnosis according to DSM V	3 (1-8)	8 (2-8)	0.19
- Former failure of withdrawal cure	6 (5-8)	3 (1-6)	0.004
- Polyaddiction	6 (3-7)	4 (2-7)	0.39
- Psychiatric comorbidities	4 (3-7)	5 (3-6)	0.97
- Socio-professional insertion	4 (3-5)	4 (4-6)	0.28
- Relatives' quality	5 (4-6)	4 (3-6)	0.79
- Insight of their alcohol use disorder	4 (1-7)	3 (2-6)	0.96
- Addictologist follow-up	5 (2-7)	6 (5-7)	0.08
- **Which score(s) do you routinely use for your addictology assessment?**			
- AUDIT score	14 (87.5)	19 (90.5)	0.77
- HRAR score	3 (18.8)	3 (14.3)	0.72
- SALT score	1 (6.3)	2 (9.5)	0.72
**In which circumstances do you support your addictology assessment by questioning the relatives?**			
- Always	15 (93.8)	13 (61.9)	0.03
- In case of patient's encephalopathy/intubation	1 (6.2)	9 (42.9)	0.01
**Who is questioned in the family when this is done?**			
- The partner	16 (100)	20 (95.2)	0.38
- Children and parents	16 (100)	20 (95.2)	0.38
- Friends /colleagues	4 (25.0)	2 (9.5)	0.21
- The general practicioner	13 (83.8)	18 (85.7)	0.72
**How often do you perform blood, urinary or capillary screening tests?**			
- < 25% patients<br>- 26 -50% patients<br>- 51 -75% patients	9 (56.3)<br>3 (18.7)<br>0 (0)	13 (61.9)<br>1 (4.8)<br>1 (4.8)	0.48

- > 75% patients	4 (25.0)	6 (28.6)	
**Which biological or urinary test is most often performed in your center?**<br>- Carboxydate Deficience Transferrine<br>- Phosphatydyléthanol<br>- Urinary ethyl glucuronide<br>- Capillary ethyl glucoronide	 <br>5 (31.2)<br>6 (37.5)<br>4 (25.0)<br>1 (6.3)	 <br>7 (33.3)<br>10 (47.6)<br>3 (14.3)<br>1 (4.8)	 <br><br><br>0.84
**Do you always take into account the addictologist's opinion?**<br>- Yes<br>- No	 <br>12 (75.0)<br>4 (25.0)	 <br>15 (71.4)<br>6 (28.6)	 <br>0.81
**Oncologic assessment**<br>**In patients above 50 years old, what exams do you perform?**			
**When do you perform a colonoscopy**<br>- In all patients<br>- in case of risk factors of colorectal neoplasm	 1 (6.3)<br>4 (25.0)	 6 (28.6)<br>5 (23.8)	 0.09<br>0.93
**Do you systematically perform an upper GI endoscopy?**<br>- Yes<br>- No	 <br>11 (68.8)<br>5 (31.2)	 <br>16 (76.2)<br>5 (23.8)	 <br>0.61
**Do you systematically perform a CT scan of the abdomen and pelvis?**<br>- Yes<br>- No	 <br>11 (100)<br>0 (0)	 <br>21 (100)<br>0 (0)	 <br>1
**Do you systematically perform a PSA dosage in men?**<br>- Yes<br>- No	 <br>11 (68.8)<br>5 (31.2)	 <br>16 (76.2)<br>5 (23.8)	 <br>0.61
**Do you systematically perform a mammography, pap smear, and gynecologic exam for women?**<br>- Yes<br>- No	 <br>5 (31.2)<br>11 (68.8)	 <br>7 (33.3)<br>14 (66.7)	 <br>0.89
**Do you systematically perform an ENT consultation?**<br>- Yes	 <br>6 (37.5)	 <br>10 (47.6)	 <br>0.54

- No	10 (62.5)	11 (52.4))	
**Do you systematically perform a dermatology consultation?**<br>- Yes<br>- No	 <br>3 (18.8)<br>13 (81.2)	 <br>5 (23.8)<br>16 (76.2)	 <br>0.71
**Nutritional assessment**			
**When do you request nutritionist or dietitian consultation?**<br>- Systematically<br>- In case of clinical malnutrition<br>- Never	 <br>7 (43.7)<br>5 (31.3)<br>4 (25.0)	 <br>8 (38.1)<br>6 (28.6)<br>7 (33.3)	 <br><br>0.86
**When do you perform a screening for malnutrition using questionnaires such as SGA, RFH-NPT**<br>- Systematically<br>- In case of clinical malnutrition	 <br>2 (12.5)<br>1 (6.3)	 <br>6 (28.6)<br>4 (19.1)	 <br>0.24<br>0.26
**When do you perform a screening for sarcopenia using psoas measurement by CT scan?**<br>- Systematically<br>- In case of clinical malnutrition	 <br>3 (18.8)<br>3 (18.8)	 <br>0 (0)<br>3 (14.3)	 <br>0.04<br>0.71
**When do you perform a screening for frailty using dedicated tests (for example LFI)**?<br>- Systematically<br>- In case of clinical malnutrition	 <br>3 (18.8)<br>3 (18.8)	 <br>2 (9.5)<br>1 (4.8)	 <br>0.42<br>0.17
**General considerations**			
**In your experience, what is the timeframe in days between initiation of LT assessment and listing?**	5 (3-7)	10 (7-15)	<0.0001
**What proportion of ACLF patients develop ACLF while already listed for LT, with a complete pre-transplant workup?**<br>- < 25% patients<br>- 26 -50% patients<br>- 51 -75% patients<br>- 75% patients	 <br><br>9 (56.3)<br>6 (37.5)<br>1 (6.25)<br>0 (0)	 <br><br>17 (81.0)<br>3 (14.3)<br>1 (4.7)<br>0 (0)	 <br><br><br>0.24

**How do you describe the pre-transplant workup in ACLF patients in ICU, as compared with other situations?**			
- Similar to other situations	1 (6.2)	0 (0)	0.55
- Abbreviated workup to allow for a quick listing assuming that it will still be able to identify definitive contraindications to LT	11 (68.8	15 (71.4)	
- Abbreviated workup to allow for a quick listing with the risk of not identifying definitive contraindications to LT	4 (25.0)	5 (23.8)	
- More comprehensive workup to secure LT results in this very sick population	0 (0)	1 (4.8)	

Abbreviations : ACLF: Acute On Chronic Liver Failure, AUD: alcohol use disorder, AUDIT: Alcohol Use Disorders Identification Test, CCAS: coronary artery calcium scan, CCTA: coronary CT angiography, DSM: Diagnostic and Statistical Manual of Mental Disorders, ECG: electrocardiogram, EGD: oesophagograstroduodenoscopy, ENT: Ear-Nose-Throat HRAR: High-Risk Alcoholism Relapse, Hosp: hospitalized patients without ALCF; LFI: liver frailty index, LT: liver transplantation, OutPat: outpatients, PSA: Prostatic Specific Antigen, RFH-NPT: Royal Free Hospital nutritional prioriziting tool, SALT: Sustained Alcohol Use After Early Liver Transplant, SGA: subjective global assessment, TAP: thoraco-abdomino-pelvic, TTE: trans-thoracic echocardiography
